# A systematic review and meta-analysis to identify behavioural content and active ingredients of antimicrobial stewardship education and training interventions in hospital-based care settings

**DOI:** 10.1186/s13756-025-01660-0

**Published:** 2025-12-18

**Authors:** Rebecca R. Turner, Nia Coupe, Sophie Griffiths, Kate Cheng, Lucie Byrne-Davis, Laura Shallcross, Jo Hart, Stephen Rice, Hosein Shabaninejad, Nick Meader, Nawaraj Bhattarai, Fabiana Lorencatto

**Affiliations:** 1 Behavioural Science for International Health Workforce Group, The University of Manchester, Manchester, UK; 2 Division of Psychology & Mental Health, The University of Manchester, Manchester, UK; 3 Psychology Department, School of Health, Education, Policing and Science, Science Centre, Trainee Health Psychologist, Staffordshire University, Leek Road, Stoke-on-Trent, ST4 2DF UK; 4 Institute of Health Informatics, University College London, London, NW1 2DA UK; 5 Health Economics Group, Population Health Sciences Institute, Newcastle University, Newcastle upon Tyne, UK; 6 Centre for Behaviour Change, University College London, 1-19 Torrington Place, London, WC1E 7HB UK

## Abstract

**Background:**

The growing threat of antimicrobial resistance has led to efforts to improve the responsible use of antimicrobials (antimicrobial stewardship - AMS). AMS education and training is essential for providing healthcare professionals with the knowledge and skills required to change prescribing behaviours, but the design and delivery of education and training varies, and it is unclear what content, and methods make for more effective education and training. The aim of this systematic review was to apply behavioural science frameworks to specify the content of AMS education and training interventions in hospital settings to determine ‘what works’ and to evaluate their effectiveness and cost-effectiveness.

**Methods:**

We searched MEDLINE, EMBASE, and CENTRAL and hand searched studies included in a previous Cochrane review for studies published from January 2015 to February 2025. We applied behavioural science frameworks (Action, Actor, Context, Target and Time framework, Behaviour Change Wheel and Behaviour Change Technique Taxonomy) to code intervention descriptions and supplementary materials from published papers into target behaviours, modes of delivery and behaviour change strategies used. Meta-regressions were used to explore the (cost-)effectiveness of different target behaviours, modalities, and behaviour change strategies on reducing antibiotic consumption.

**Results:**

Of the 1845 studies identified, 64 were included in the review and 26 included in the meta-regression. Education/training was more effective in reducing antibiotic consumption when delivered face-to-face (β= − 2.65, 95% CI: − 5.23 to − 0.07, k = 21). In total, 29 behaviour change techniques were identified across interventions, with no individual behaviour change technique associated with reduced antibiotic consumption. Interventions using the broad intervention types of *modelling* (Providing an example for people to aspire to or imitate) (β= − 2.23 (95% CI: − 4.27 to − 0.18) and *restriction* (Using rules to reduce the opportunity to engage in the target behaviour or to increase the target behaviour by reducing the opportunity to engage in competing behaviours) (β = 2.95 (95% CI: 1.10 to 4.79) had significant effects on antibiotic consumption.

**Conclusion:**

Our results suggest that AMS education and training interventions may be more effective when they focus on modelling and appropriate restriction, and when delivered in-person. However, more evidence is needed from well-designed studies that explicitly report intervention content, to enable firmer conclusions about the specific elements involved in effective AMS education and training.

**Supplementary Information:**

The online version contains supplementary material available at 10.1186/s13756-025-01660-0.

## Background

Antimicrobial resistance (AMR) is one of the leading global health threats (ESPAUR 2024; Smith et al., 2020), with an estimated 4.71 million deaths associated with bacterial AMR in 2021 (Naghavi et al., 2024). One key cause of AMR is the overuse of antibiotics in hospital-based care (Gulliford et al. 2020), which is currently rising year on year (ESPAUR 2024). Healthcare professionals play a critical role in the development and prevention of AMR through their prescribing and infection control practices. Antimicrobial stewardship (AMS) programmes within healthcare settings aim to promote and monitor the judicious use of antimicrobials to preserve their future effectiveness (NICE 2015). AMS interventions aim to ensure more prudent use of antimicrobials whilst still ensuring safe and effective treatment for patients (Davey et al. 2017). AMS interventions are inherently complex due to AMS itself being behaviourally complex (Davey et al. 2017). For example, AMS involves multiple actions, performed at different timepoints across the whole healthcare system, these actions can range from adhering to guidelines, decision making around antibiotic initiation, drug choice or switching from intravenous antibiotics to oral antibiotics (Charani et al. 2013, 2014; Lorencatto et al. 2018; Rodrigues et al. 2013).

AMS interventions have many components such as audit and feedback, decision support and public facing campaigns, but the core components are AMS education and training (Davey et al. 2017). Education and training are typically the default intervention in healthcare to encourage healthcare professional behaviour change to implement new guidance or technologies into standard practice (Johnson and May 2015). Education aims to increase knowledge and understanding, whereas training aims to impart skills (Michie et al. 2011). AMS education and training is equally complex in comparison to other intervention functions and are often designed, delivered, implemented and evaluated in different ways across UK hospital-based care (Charani et al. 2019; Davey et al. 2017; Rzewuska et al. 2019; Turner et al. 2023). For example, often education and training approaches may be too generic (i.e., not tailored to a specific clinical context) with decisions about the content, mode of delivery and format not being based upon relevant theory or evidence (Eccles et al. 2005). This can in turn lead to vital learning objectives not being met, which may limit the effectiveness of the education and training, leading to unpredictable and potentially costly consequences for safe patient care (Eccles et al. 2005).

To learn from current research and practice successes and failures and identify opportunities to optimise AMS education and training we need to carefully examine how these initiatives have been developed, implemented, and assessed, and whether they explicitly address the behavioural determinants underpinning AMS-related behaviours.

A range of tools exist to help researchers and practitioners to apply behavioural science to gain a better understanding of what such interventions look like and how they work. These tools are being increasingly applied in systematic reviews to specify behaviour change components and target behaviours in complex interventions (Crayton et al. 2020; Kocur et al. 2024; Richardson et al. 2019). These tools include *The Behaviour Change Wheel* (BCW) (Michie et al. 2011), an intervention development framework which specifies types of interventions including education and training. *The Behaviour Change Techniques* (BCTs) taxonomy (Michie et al. 2013) that distinguishes more granular behaviour change strategies (e.g., information about health consequences and action planning). The *Actor*,* Action*,* Context*,* Target and Time framework (AACTT)* which supports detailed behaviour specification, clarifying who needs to do what differently (Presseau et al. 2019). These tools can be used to code interventions to identify and characterise the components of complex interventions, such as AMS education and training, to help create a shared language between researchers, improve reporting and therefore our understanding of what might work or what might not. It is also important to note that interventions may still act on relevant behavioural influences even if they were not explicitly informed by theory. This justifies the application of theoretical frameworks retrospectively to analyse and interpret existing interventions, as has been done in other systematic reviews. Such approaches allow for a structured understanding of the underlying mechanisms of action, even when original authors have not made these explicit. These tools also provide a basis for comparing and exploring associations with outcomes via meta-regression as they allow the extraction of standardised concepts that can be examined as moderators of intervention effectiveness. This facilitates comparison across diverse studies and supports the identification of components associated with improved outcomes, offering insights into both efficacy and underlying mechanisms of action.

This review aimed to systematically synthesise published AMS education and training interventions in hospital-based care using behavioural science frameworks to evaluate effectiveness and cost-effectiveness.

The five distinct objectives were as follows:

1. To identify whose and which behaviours have been targeted by published AMS education and training interventions.
2. To identify which modes of delivery have been used to deliver AMS education and training in hospital-based care.
3. To identify which intervention types and BCTs make up the content of AMS education and training in hospital-based care.
4. To understand how the effectiveness of AMS education and training in hospital-based care varies according to which/whose behaviours are targeted, modes of delivery, intervention types and BCTs used.
5. To understand how cost-effective AMS education is and training and is there evidence that any intervention components or modes of delivery are more likely to be part of cost-effective interventions than others.

## Methods

### Design

This systematic review was conducted in accordance with the Preferred Reporting Items for Systematic Reviews and Meta-Analyses (PRISMA) guidelines (Moher et al. 2009). The systematic review protocol was prospectively registered on PROSPERO (CRD42023405711) (Lorencatto et al. 2023).

### Search strategy

Our search strategy mirrored the published search strategy of the Cochrane Review of a broad range of AMS interventions in hospital-based care (Davey et al. 2017), with minor adaptations to use a narrower range of terms related to our specific focus on AMS education and training interventions. We structured the search terms around the target problem (antimicrobial resistance/ AMS), intervention (education and training), setting (hospital-based care) and study designs (randomized and non-randomized trials). The full search strategy is available in Supplementary file 1.

We searched three electronic databases (MEDLINE, EMBASE, and Cochrane Central Register of Controlled Trials (CENTRAL)) and hand searched studies included in the Cochrane review. The search date range was from January 2015 (date of the last search in the Cochrane review) to October 2022 and updated in February 2025.

### Eligibility criteria

Our full eligibility criteria is reported in the PROSPERO registration (CRD42023405711) (Lorencatto et al. 2023). We included AMS education and/or training interventions delivered to any healthcare provider of any role and level of experience, caring for any inpatient population group(s), in any hospital-based care setting.

To best answer our research questions, we focused on interventions for which education was a substantive component of the intervention. Through consultation with our expert advisory groups, we agreed that education and/or training terms needed to be used in the title and/or abstract of the published paper to be included. Existing definitions of education and training (Michie et al. 2011) and associated activities ((EPOC), 2015) were used, see Table 1. Education and training interventions delivered by any modality were eligible for inclusion.

**Table 1:** Definitions of education, training and associated activities

Intervention type	BCW Definition (Michie et al. 2011)	EPOC Definition ((EPOC), 2015)	EPOC examples / Associated activities
Education	Increasing knowledge or understanding.	Aims to increase knowledge and understanding through the provision of information.	Educational meetings (e.g. lectures, seminars) Distribution of educational materials (e.g. guidelines, printed or digital) Educational outreach visits (with an informational focus)
Training	Imparting skills.	Aims to develop healthcare professionals’ skills, often through practice or simulation, to improve performance.	Workshops or practical sessions Simulations Role-play Skills-based small group training On-the-job training or mentoring

We included both randomised designs (RCTs, cluster-RCTs), and non-randomised designs (controlled before and after studies, and interrupted time series). The comparator was any other comparator group, which could include another intervention or no-treatment/ usual practice control group. There was no limitation on geographical location. Studies had to be published in English, in peer-reviewed journals.

### Screening

Search results up until October 2022 were imported into Rayyan software for de-duplication and title and abstract screening, and subsequently full-text screening against the eligibility criteria. Both stages were completed independently by two reviewers (SG, KC), with any discrepancies discussed with additional team members (FL, LS) to reach consensus. The search was updated from October 2022 to February 2025, and these were managed in Endnote. Both stages were completed independently by two reviewers (RT, NC), with any discrepancies discussed with additional team members (FL, LBD) to reach consensus. We applied the same inclusion/exclusion criteria to the studies included in a previous Cochrane review of AMS interventions in hospital-based care (Davey et al. 2017) and included studies from this review that were eligible.

### Data extraction and analysis

We co-developed our data extraction form with our key stakeholder groups: (1) Patient and public involvement group, (2) Policy and practice stakeholders and (3) Academic advisory group. Two independent reviewers extracted the data (SG, KC) and (NC, RT for the updated search), with discrepancies discussed and agreed with a third author (FL, LS, LBD, NM). Our full data extraction is reported in the PROSPERO registration (Lorencatto et al. 2023). We present the following data items for this manuscript, these are as follows; extracted publication details (author(s), title, date, country), study characteristics (design, type and number of hospitals/ wards, duration, length of follow-up, method and frequency of data collection), delivery characteristics (mode of delivery, frequency, duration, tailoring, use of theory, the providers and recipients of the intervention), intervention outcomes (primary outcome - antibiotic consumption, secondary outcome - appropriateness of prescribing, infection rates). We used narrative synthesis and descriptive statistics to summarise data on general study characteristics (i.e. country, year, sample size, study design).

To identify and specify the AMS behaviours targeted by the interventions (RQ1), we used the coding framework ‘Action, Actor, Context, Target, Timing’ (AACTT; Presseau et al. 2019).

To identify which modes of delivery have been used to deliver AMS education and training (RQ2) we descriptively summarised extracted data on mode of delivery parameters, including modality of delivery (i.e. face to face, electronic, printed, hybrid), frequency, duration, and source/provider.

To identify which intervention types and BCTs make up the content of AMS education (RQ3), we used the BCTTv1 taxonomy as a coding framework (Michie et al. 2013) alongside illustrative examples to identify behaviour change techniques (BCTs) within the interventions. The BCW (Michie et al. 2011) was used to code intervention functions (means by which an intervention can change behaviour). Inter-rater reliability of BCT coding was calculated using Krippendorff’s alpha (α) reliability coefficient (Hayes & Krippendorff, 2007) for a sub-set (33%) of included papers each coded by two reviewers. We extracted the source (provider), the frequency (how often sessions occurred), the duration (total intervention length), and the modality (face-to-face, telephone, email, website, electronic or printed materials) of interventions.

### Meta-regression analysis

To understand how the effectiveness of AMS education and training in hospital-based care vary according to which/whose behaviours are targeted, modes of delivery, intervention types and BCTs used (RQ4), we extracted measurement of treatment effects. For RCTs and controlled before-after studies, we extracted the odds ratio (OR) or relative risk (RR) relating to intervention effectiveness with 95% confidence intervals. We used post-intervention statistic from studies to standardise the effect measured and to ensure consistency in our review of the studies. Systematic reviews including studies with primary ITS data often require further analyses. Estimates based on appropriate methods for analysing ITS data were extracted from included studies. However, when either insufficient data were reported, or the methods were not judged to be appropriate, we re-analysed the data using segmented regression methods when graphs reported at least 5 data points before and after the intervention. We used Graph Grabber 2.0.2 software to estimate data from graphs and then conducted segmented regression analyses on these data. We also conducted Prais-Winsten adjustment to reduce the risk of overfitting. Digitizing graphs can sometimes lead to extreme values, therefore we conducted sensitivity analyses removing potential outliers. Meta-regressions were then conducted to assess the effect of characteristics and components of AMS education and training interventions on outcomes. These analyses were only carried out for interventions that measured changes in antibiotic consumption. Heterogeneity across studies in the measurement of other outcomes meant there was insufficient data for us to perform meta-regressions for other outcomes. Covariates examined included:

- Target behaviour categorised dichotomously as whether the intervention targeted an increase in an AMS behaviour (i.e. increased documentation of prescribing decisions, increased reviewing) vs. a reduction (i.e. reduced prescribing); target context, categorised as targeting multiple vs. single wards/clinical specialties.
- Mode of delivery (print, telephone, email, web, face-to-face, electronic); source, categorised as either AMS specialist (e.g. AMS pharmacist, infectious disease physician, microbiologist), or general HCP (e.g. surgeon).
- BCW intervention types, and individual component BCTs.

Meta-regression analyses (with studies included irrespective of risk of bias scores) with backward elimination (all covariates entered in model, covariates with high p-values (*p* > 0.15) removed) were conducted. Associations between remaining covariates and outcome were assessed. Statistical significance for covariates in final models was set at *p* < 0.05. We also examined potential interactions between covariates using the meta-classification and regression trees (meta-CART) R-package. This method is particularly useful when investigating many covariates and therefore a higher number of potential interactions. The meta-CART package uses classification and regression trees to identify the best combination of predictors of outcome by “pruning” those potential interactions that are not associated with outcome. We used regression trees with random effects weights. We used the minimum cross-validation rule which minimizes squared difference between the observed and predicted values of outcome (c = 0) but also assessed whether varying c (minimum rule + 0.5 or 1 standard error) impacted results. For both meta-regressions and meta-cart analyses, we included all covariates with at least two studies for each category. We also conducted sensitivity analyses with at least five studies per category and removing potential outliers. Given the very large number of covariates assessed, these meta-regression analyses are inherently exploratory.

### Risk of bias

Risk of bias was applied to the studies that measured the primary outcome (antibiotic consumption) only, as per Cochrane guidance (Boutron 2023), using the risk of bias in randomised trials (RoB2) (Sterne et al. 2019) and non-randomised trials (ROBINS-I)(Sterne et al. 2016) tools. For interrater consistency, all risk of bias assessments were conducted by two reviewers, with discrepancies resolved by discussion with a third reviewer (FL, LS, RT, NC).

### Cost-effectiveness analysis

To understand how cost-effective the AMS education and training is and examine the evidence that any intervention components or modes of delivery are more likely to be part of cost-effective interventions than others (RQ5), a cost-effectiveness analysis was undertaken. A detailed description of the methods of the health economics is provided in Supplementary file 2. The health economics analysis was only conducted for studies included in the meta-regression effectiveness analyses. Key resource utilisation components of activities related to the design and delivery of the intervention were extracted from published intervention reports (e.g. number of hours required to produce the intervention, number of staff involved in delivering/receiving the intervention, duration of educational sessions, etc.).

Unit costs for these resources were extracted from the literature or obtained through other relevant sources such as NHS reference costs, the PSSRU and manufacturer price lists.32,33 The total cost of the intervention in each study was estimated. Scenario analyses were conducted on the total cost calculations where values used in assumptions were varied. The measure of benefit was the relative treatment effect measure reported above in the effectiveness meta-regressions.

The incremental costs associated with the explanatory factors in each of 4 sets of covariates (BCT [Objective 3], BCW [Objective 3], mode of delivery [Objective 2], context [Objective 1]) were estimated using generalised linear models. The corresponding incremental benefits were obtained from the meta-regression effectiveness analyses. The incremental cost and benefit associated with each explanatory factor for each set of covariates was presented in a cost-effectiveness plane, the North-East quadrant is associated with greater effectiveness but also greater cost. The strength of evidence of the effectiveness and cost difference association (positive or negative) were simply represented using differently coloured letters for different combinations of p-values for cost and effect coefficients.

## Results

### Included studies

**Fig. 1:**
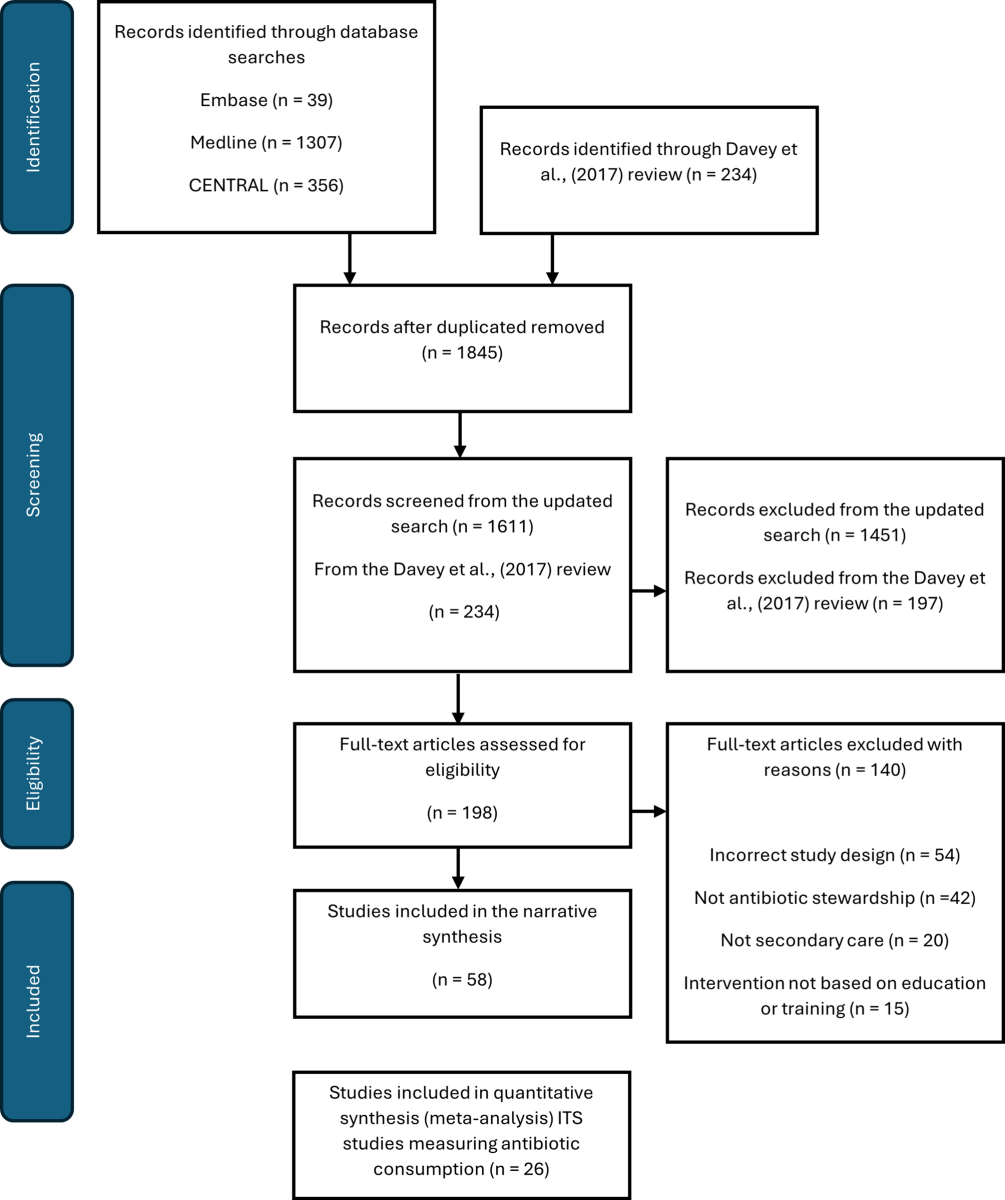
PRIMSA

The search results are summarised in a PRISMA diagram in Fig. 1. We screened 1702 results for inclusion from electronic database searches. Following de-duplication and screening against inclusion criteria 36 studies were included. A further 23 studies were identified as meeting the inclusion criteria via the hand search of studies in the original Cochrane review (Davey et al. 2017), resulting in a total of 58 studies being included. These studies reported 64 AMS education and training interventions in total.

### Summary of study characteristics

Study characteristics are summarised in Table 2. In summary, studies were published between 1988 and 2024 and were predominantly conducted in high-income countries (*n* = 52, 93.10%). Most studies used an interrupted time-series design (*n* = 43, 74.1%), followed by randomised controlled trials (*n* = 10, 17.24%). A minority of studies carried out prior formative work (*n* = 11, 19.0%) or explicitly used any theory (*n* = 7, 12.7%) to inform intervention design. Most studies measured intervention effectiveness using antibiotic consumption (*n* = 26, 47.2) in Defined Daily Doses (DDDs) or the duration of treatment in days.

**Table 2:** Summary of study characteristics

First author (year)	Country	Trial Design	Setting	Number of interventions	Number of comparator conditions	Use of theory/ framework	Outcomes	Sample size	Study duration	Follow-up	Risk of bias
Adachi et al. (1997)	USA	ITS	Community hospital	1	0	None	Antibiotic consumption (DOTs)	Not reported	36 months	21 months	Low
Alvarez-Marin et al. (2021)	Spain	ITS	University hospitals, specialty hospitals	1	0	None	Antibiotic consumption (DDDs); Infection rate (C. Difficile)	Not reported	45 months	24 months since introduction of intervention	Critical
Avorn et al. (1988)	USA	ITS	Teaching hospital	1	0	Prior formative work: literature review	Appropriateness	Not reported	39 months	19 months	
Barbieri et al. (2020)	Italy	CITS	Tertiary and secondary-level university hospitals	2	1	None	Antibiotic consumption (DOTs)	Not reported	Centre A: 42 months Centre B: 30 months Centre C: 42 months	35 months	Moderate
Belliveau (1996)	USA	ITS	University hospital	1	0	None	Antibiotic consumption (DOTs)	Not reported	16 months	4 months	Low
Buising et al. (2008)	Australia	ITS	University hospital	1	1	None	Appropriateness (% concordant with recommendations)	30	41 months	Academic detailing: 9 months Computerised decision support: 6 months	
Camins et al. (2009)	USA	RCT	Public university-affiliated teaching hospital	1	1	None	Appropriateness	66	10 months	10 months	
Carrara et al. (2022)	Italy	ITS	Tertiary care hospital	1	0	Behavioural theory: the Behaviour Change Wheel	Antibiotic consumption (DDDs, DOTs); Infection rates (C. Difficile, Carbapenem-resistant enterobacterales BSIs)	104	36 months	9 months	Low
Chang et al. (2017)	Taiwan	ITS	University hospital	1	1	None	Antibiotic consumption (DDDs)	Not reported	18 months	6 months	Critical
Corcione et al. (2022)	Italy	ITS	Tertiary care teaching hospital	1	0	None	Antibiotic consumption (DDDs)	Not reported	48 months	30 months	Critical
Daly et al. (2022)	Australia	ITS	Surgical department in hospital	1	0	None	Antibiotic consumption (DOTs, unanalysable); Infection rate (SSIs)	Not reported	73 months	15 months	Critical
Danaher et al. (2009)	USA	RCT	Military teaching hospital	1	1	Other	Antibiotic consumption (DDDs)	Not reported	6 months	6 months	High
Du et al. (2020)	China	ITS	Teaching hospital	1	0	None	Antibiotic consumption (DDDs)	Not reported	36 months	18 months	Low
Everitt et al. (1990)	USA	ITS	University hospital	1	0	Behavioural theory: the Behaviour Change Wheel Prior formative work: staff interviews	Prophylaxis:	Not reported	34 months	24 months	
Flett et al. (2018)	USA	ITS	Children’s hospital	1	1	Prior formative work: chart review	Antibiotic consumption (	56	27 months	9 months	Critical
Fralick et al. (2017)	Canada	Controlled before-and-after	Tertiary care teaching hospital	1	1	None	Knowledge (scores)	32	5 months	30 days	
Garcia-Martinez et al. (2016)	Spain	ITS	Tertiary university hospital	1	0	Prior formative work: literature review	Antibiotic consumption (DDDs); Infection rate (Linezolid-resistant bacteria).	Not reported	36 months	23 months	Serious
Gardiner et al. (2018)	New Zealand	ITS	Public hospitals	1	0	Prior formative work: literature review	Antibiotic consumption (DDDs)	Not reported	96 months	48 months	Some concerns
Gardiner et al. (2020)	New Zealand	ITS	Public hospitals	1	0	Behavioural theory: the Behaviour Change Wheel	Antibiotic consumption (DDDs)	Not reported	83 months	24 months	Critical
Hadi et al. (2008)	Indonesia	ITS	Teaching hospital	1	0	None	Antibiotic consumption (DDDs); Appropriateness (% concordant with guideline)	83	13 months	6 months	Critical
Huang et al. (2018)	Taiwan	ITS	Tertiary ICU	1	0	Prior formative work: literature review	Prophylaxis antibiotic consumption (DDDs); Appropriateness (% concordant with guideline); Infection rate (resistant bacteria)	Not reported	90 months (June 2002-Dec 2009)	N/A- only baseline and during intervention data is provided	Some concerns
Irfan et al. (2015)	Canada	Controlled before-and-after	Tertiary teaching adult care hospitals	1	1	Prior formative work: review of risk factors at institution	Appropriateness (unnecessary use)	Not reported	14 months (Feb 2012- April 2013)	23 months (urine cultures ordered on intervention unit), 26 months (patients with ABU unecessarily treated)	
Itoh et al. (2023)	Colombia	ITS	Tertiary hospitals	1	0	None	Antibiotic consumption (DOTs)	Not reported	60 months	24 months	
Kashtan et al. (2020)	USA	ITS	Children’s hospital	1	0	Behavioural theory: Plan–Do–Study–Act framework	Appropriateness (compliance with guidelines); Infection rates (SSIs)	Not reported	15 months	10 months	
Kishida and Nishiura (2020)	Japan	CITS	Teaching hospitals	1	0	Prior formative work: literature review	Infection rates (resistant bacteria)	Not reported	120 months	3 months	
Kjaersgaard et al. (2019)	Denmark	ITS	Tertiary hospitals	2	0	Other	Antibiotic consumption (DDDs); Appropriateness (% concordant with guideline)	288	24 months	Hospital A-4 months. Hospital B- 0 months	Critical
Knudsen et al. (2014)	Denmark	CITS	University hospital	1	1	Behavioural theory: Kotter’s 8-step change model	Antibiotic consumption (DDDs); Infection rates (resistant bacteria, C. Difficile)	Not reported	48 months	24 months	Critical
Kushala et al. (2023)	Japan	ITS	Children’s hospital	1	1	Prior formative work: root-cause analysis to understand factors that influenced non-adherence to guidelines	Appropriateness (compliance with guidelines)	98	6 months	0 months	
Landgren et al. (1988)	Australia	Controlled before-and-after	Metropolitan teaching hospitals, suburban general hospital, rural hospitals	1	1	None	Prophylaxis appropriateness (assessed for duration, timing)	Not reported	17 months	12 months	
Lee (2014)	Canada	ITS	University/ tertiary care hospital	1	0	None	Antibiotic consumption (DDDs); Infection rate (C. Difficile)	38	38 months	16.5 months	Low
Liebowitz and Blunt (2008)	England	ITS	General, acute hospital	1	0	None	Antibiotic consumption (DDDs); Infection rate (resistant bacteria)	Not reported	36 months	16 months	Low
Liu et al. (2021)	China	ITS	Tertiary teaching hospital	1	0	None	Antibiotic consumption (DDDs)	Not reported	3 months	12 months	Moderate
McLellan et al. (2016)	England	RCT	Teaching hospital	1	1	None	Appropriateness (compliance with criteria)	14	6 months (Jan 2013-July 2013)	Not reported	High
Mol et al. (2005)	the Netherlands	ITS	University hospital	1	0	None	Appropriateness (compliance with guidelines)	Not reported	36 months	9 months	
Molina et al. (2017)	Spain	ITS	Tertiary care teaching university hospital	1	0	None	Antibiotic consumption (DDDs); Infection rates (resistant bacteria, hospital-acquired candidemia)	Not reported	28 quarters (84 months) between Jan 2009-December 2015	0 months	Critical
Molina et al. (2019)	Spain	ITS	Tertiary care university hospital	1	0	None	Antibiotic consumption (DDDs); Infection rates (pathogens in bloodstream infections)	Not reported	102 months	0 months	Low
Perez et al. (2003)	Colombia	ITS	University hospital	1	0	Prior formative work: focus groups	Appropriateness (incorrect use)	Not reported	34 months	38 weeks	
Popovski et al. (2015)	Canada	ITS	Tertiary care teaching hospital	1	0	None	Antibiotic consumption (DDDs)	Not reported	19 months	24 months	Low
Roger et al. (2020)	France	Controlled before-and-after	Private hospitals	1	1	None	Antibiotic consumption (DDDs); Appropriateness (unnecessary use)	23	12 months	9 months	Critical
Saizy-Callaert et al. (2003)	France	ITS	General hospital	1	0	None	Antibiotic consumption (expenditure)	Not reported	48 months	4 years	Critical
Schwartz et al. (2017)	USA	RCT	Urban teaching hospital	1	0	None	Appropriateness (DOT with error)	Not reported	5.5 months	0 months	High
Schwartz et al. (2007)	USA	ITS	Public long-term care and acute care hospital	2	1	None	Antibiotic consumption (DOTs)	20	4.5 years	5 months	Critical
Schweitzer et al. (2022)	the Netherlands	CRCT	University, teaching, and non-teaching hospitals	1	1	Prior formative work: literature review	Antibiotic consumption (DOTs)	235	21 months	3 months	Some concerns
Seddik et al. (2021)	USA	ITS	Quaternary-care academic medical centre hospital	1	0	None	Antibiotic consumption (DOTs); Infection rate (SSIs, C. Difficile)	Not reported	42 months	24 months	Critical
Skaer et al. (1993)	USA	ITS	Rural acute care hospital	1	0	None	Antibiotic consumption (DOTs)	Not reported	23 months (Jan 1991-Dec 1992)	18 months	Low
Smoke et al. (2022)	USA	ITS	Teaching hospital	1	0	None	Antibiotic consumption (DOTs); Infection rates (susceptibility)	Not reported	22 months	10 months	Critical
Solomon et al. (2001)	USA	CRCT	Teaching Hospital	1	1	None	Appropriateness (unnecessary prescriptions)	Not reported	22 weeks (5.5 months) (study period was 20.01–19.05 1999 and the 4 weeks preceding this were considered the baseline)	0 months	
Spoorenberg et al. (2015)	The Netherlands	CRCT	University and non-university hospitals	1	1	Behavioural theory: improvement strategy developed my Schouten et al. (2007)	Appropriateness (compliance with criteria)	Not reported	47 months (Feb 2009-Jan 2013)	10 months	
Standiford et al. (2012)	USA	ITS	Tertiary Care University Teaching Medical Centre	1	0	None	Antibiotic consumption (expenditure)	Not reported	10 years (120 months) (2001–2010)	24 months	Critical
Stenehjem et al. (2018)	USA	CRCT	Small hospitals part of a non-profit healthcare delivery system	2	1	None	Antibiotic consumption (DOTs)	Not reported	53 months (baseline: Jan 2011-June 2013, implementation: July 2013-March 2014, intervention: April 2014-June 2015)	0 months	Some concerns
Tang et al. (2019)	USA	NRS	Urban, tertiary care teaching hospital	2	1	Behavioural theory: a driver diagram based on a model for improvement Prior formative work	Antibiotic consumption (DOTs)	Not reported	15 months (intervention September-Dec 2016) -historic data from the corresponding months in Sept-Dec 2015 were compared and used as the baseline period	0 months	Critical
Tangden et al.(2011)	Sweden	ITS	Tertiary university hospital	1	0	None	Antibiotic consumption (DDDs); Infection rates (resistant bacteria)	Not reported	8 years (96 months)	2.5 years	Low
Tedeschi et al. (2017)	Italy	ITS	Rehabilitation hospital	1	0	None	Antibiotic consumption (DDDs); Infection rates (resistant bacteria)	Not reported	35 months (before: 1 Jan 2011- 30 June 2012, after: 1 July 2012-31 December 2014)	0 months	Low
van Hees et al. (2008)	The Netherlands	ITS	Teaching hospital and tertiary referral centre	1	0	None	Excluded data: Appropriateness (unnecessary use)	Not reported	18 months (Sept 2004-March 2006)	8 months	
van Horrik et al. (2024)	The Netherlands	CRCT	Teaching hospitals and general district hospitals: (1 University hospital and 4 general hospitals)	5	5	None	Appropriateness (unnecessary prescriptions)	1958 participants	12 months	Not reported	
Vercheval et al. (2016)	Belgium	ITS	Academic teaching hospital (University hospital)	1	0	None	Appropriateness (compliance with criteria)	Not reported	23 months (Phase 1, Pre-intervention: May 2012-Feb 2013, Intervention: March 2013-May 2013, Phase 2, Post-intervention: June 2013-March 2014)	10 months	
Wathne et al. (2018)	Norway	CRCT	Emergency care and teaching hospitals: (2 tertiary care hospitals and 1 secondary care hospital)	2	1	None	Antibiotic consumption (DDDs); Appropriateness (compliance with guidelines)	Not reported	36 months	18 months	Some concerns
Willemsen et al. (2010)	The Netherlands	ITS	Teaching hospital	1	0	None	Antibiotic consumption (DDDs); Infection rate (resistant bacteria)	Not reported	24 months	0 months	Low

### Which behaviours were targeted by interventions?

The behaviours (i.e. AMS actions) targeted by education and training interventions specified according to the AACTT framework are presented in Table 3.

**Table 3:** Studies categorised by Target, Action, Context, timeframe and actor

First author (year)	Context(s)	Target(s)	Actor(s)	Action(s)	Timing
Adachi et al. (1997)	Hospital-wide	Patients admitted	Physicians	Appropriate prescribing of both prophylactic and treatment antibiotics; documenting	
Alvarez-Marin et al. (2021)	Hospital-wide	Patients admitted	Physicians	De-escalation: broad to narrow; reviewing; documenting; appropriate antibiotic treatment prescribing	De-escalation after 48–72 h
Avorn et al. (1988)	Hospital-wide	Patients admitted	Physicians	Appropriate antibiotic treatment prescribing; documenting	Prescribing at 8-hour dosing intervals
Barbieri et al. (2020)	Paediatric emergency	Acute otitis media; pharyngitis	Unclear	Appropriate antibiotic treatment prescribing	
Belliveau (1996)	Hospital-wide	Patients admitted	Physicians	Appropriate prescribing of both prophylactic and treatment antibiotics	
Buising et al. (2008)	Emergency	Community-acquired pneumonia	Pharmacists; medical doctors	Appropriate antibiotic treatment prescribing	
Camins et al. (2009)	Internal medicine ward	Patients admitted	Physicians	Appropriate antibiotic treatment prescribing	
Carrara et al. (2022)	Internal medicine, geriatric wards	Patients admitted	Senior physicians; junior residents	Appropriate antibiotic treatment prescribing	
Chang et al. (2017)	Hospital-wide	Patients admitted	Primary prescribers; Physicians	Appropriate antibiotic treatment prescribing	
Corcione et al. (2022)	Internal medicine, surgical wards (general surgery and urology)	Patients admitted	Physicians	Appropriate prescribing of both prophylactic and treatment antibiotics	
Daly et al. (2022)	Surgical ward	Surgical patients	Surgeons; residents	Appropriate antibiotic prophylaxis prescribing	Prescribe for up to 24 h post-operation
Danaher et al. (2009)	Hospital-wide	Patients admitted	Physicians	Appropriate antibiotic treatment prescribing; de-escalation: switching from intravenous to oral antibiotics	
Du et al. (2020)	Gastroenterology ward	Patients admitted	Physicians; patients	Appropriate antibiotic treatment prescribing	
Everitt et al. (1990)	Obstetrics & gynaecology ward	Patients admitted	Physicians	Appropriate antibiotic prophylaxis prescribing	Prescribe by administering < 8 h dose intervals
Flett et al. (2018)	Paediatric gastroenterology ward, hospital-wide	Intraabdominal infections	Attending and trainee physicians	Appropriate antibiotic treatment prescribing	
Fralick et al. (2017)	Internal medicine	Patients admitted	Residents; students	Appropriate antibiotic treatment prescribing	
Garcia-Martinez et al. (2016)	Hospital-wide	Patients admitted	Attending physicians	Appropriate antibiotic treatment prescribing	
Gardiner et al. (2018)	Hospital-wide	Community-acquired pneumonia	Prescribers	Appropriate antibiotic treatment prescribing; de-escalation: switching from intravenous to oral antibiotics	
Gardiner et al. (2020)	Hospital-wide	Patients admitted	Unclear	Appropriate antibiotic treatment prescribing; de-escalation: switching from intravenous to oral antibiotics	Prescribe by administering twice daily dosing orally, or 12-hourly intravenously
Hadi et al. (2008)	Internal medicine	Fever	Residents	Appropriate antibiotic treatment prescribing; de-escalation: stopping	Stop antibiotics after 72 h
Huang et al. (2018)	ICU	Surgical patients	Prescribing physicians	Appropriate prescribing of both prophylactic and treatment antibiotics	Prescribe within 48 h of incision
Irfan et al. (2015)	Internal medicine	Asymptomatic bacteriuria	Residents; nurses	Appropriate antibiotic treatment prescribing	
Itoh et al. (2023)	Surgical ward	Patients admitted	Physicians; nurses; pharmacists	Appropriate antibiotic treatment prescribing	
Kashtan et al. (2020)	Surgical ward	Surgical patients	Attending surgeons; attending residents	Appropriate antibiotic prophylaxis prescribing	Prescribe within 60 min of incision
Kishida and Nishiura (2020)	Hospital-wide	Patients admitted	Physicians	Appropriate antibiotic treatment prescribing	
Kjaersgaard et al. (2019)	Emergency, ICU, Obstetrics & gynaecology, surgical ward, medical ward,	Patients admitted	Senior physicians; junior physicians	Appropriate antibiotic treatment prescribing	
Knudsen et al. (2014)	Hospital-wide	Patients admitted	Physicians; nurses; pharmacists	Appropriate prescribing of both prophylactic and treatment antibiotics; documenting	Prescribe by administering once before orthopaedic surgery (three doses on the day of alloplastic implantations), and before pacemaker-implantations.
Kushala et al. (2023)	One of three paediatric medical units	Patients admitted	Resident doctors	Appropriate antibiotic treatment prescribing	
Landgren et al. (1988)	Hospital-wide	Surgical patients	Surgeons; medical doctors	Appropriate antibiotic prophylaxis prescribing	Administer < 1 h after the commencement of surgery, continued for up to 24 h after surgery for non-implantation surgery and for 48 h after surgery for implantation surgery
Lee et al. (2014)	Internal medicine	Patients admitted	Junior residents; senior residents	Reviewing; appropriate antibiotic treatment prescribing	Review at the end of each month
Liebowitz and Blunt (2008)	ICU	Surgical patients	Medical doctors	Appropriate prescribing of both prophylactic and treatment antibiotics	
Liu et al. (2021)	Hospital-wide	Patients admitted	Physicians; patients	Appropriate prescribing of both prophylactic and treatment antibiotics	
McLellan et al. (2016)	Hospital-wide	Patients admitted	Junior doctors	Appropriate prescribing of both prophylactic and treatment antibiotics; documenting	
Mol et al. (2005)	Internal medicine, gastroenterology, haematology, nephrology wards	Patients admitted	Physicians	Appropriate prescribing of both prophylactic and treatment antibiotics	
Molina et al. (2017)	Hospital-wide	Patients admitted	Prescribers	Appropriate antibiotic treatment prescribing	
Molina et al. (2019)	Oncology	Oncology patients	Medical doctors	De-escalation: not clear, switching from intravenous to oral antibiotics; appropriate antibiotic treatment prescribing	
Perez et al. (2003)	Obstetrics & gynaecology, surgical ward, ICU, paediatric department	Patients admitted; surgical patients	Physicians; surgeons	Documenting; appropriate prescribing of both prophylactic and treatment antibiotics	For treatment antibiotic prescriptions: Administer dose at intervals of every 24, 6 and 8 h. respectively for aminoglycosides, first-generation cephalosporins and third generation cephalosporins. For prophylactic antibiotic prescriptions: Administer within 1 h before surgical incision
Popovski et al. (2015)	Surgical ward	Intraabdominal infection	Residents; surgical staff	Appropriate antibiotic treatment prescribing; de-escalation: switching from intravenous to oral antibiotics	
Roger et al. (2020)	Medical ward, surgical wards	Patients admitted	Physicians	Appropriate antibiotic treatment prescribing	
Saizy-Callaert et al. (2003)	Hospital-wide	Patients admitted; surgical patients	Prescribing physicians	Appropriate antibiotic treatment prescribing	
Schwartz et al. (2017)	Internal medicine	Patients admitted	Resident and attending physicians	Appropriate antibiotic treatment prescribing	
Schwartz et al. (2007)	Hospital-wide	Patients admitted	Physicians	Documenting; appropriate antibiotic treatment prescribing, de-escalation: broad to narrow	
Schweitzer et al. (2022)	Internal medicine, pulmonary wards	Community-acquired pneumonia	Physicians	Appropriate antibiotic treatment prescribing, de-escalation: broad to narrow, switching from intravenous to oral antibiotics	
Seddik et al. (2021)	Surgical ward	Paediatric appendicitis	Surgeons	Appropriate antibiotic treatment prescribing	
Skaer et al. (1993)	Hospital-wide	Patients admitted	Prescribing physicians	Appropriate antibiotic treatment prescribing	
Smoke et al. (2022)	Hospital-wide	Patients admitted	Not reported	Appropriate antibiotic treatment prescribing	
Solomon et al. (2001)	Medical, oncology, cardiology wards	Patients admitted	Resident physicians	Appropriate antibiotic treatment prescribing	
Spoorenberg et al. (2015)	Internal medicine, urology wards	Urinary tract infections	Not reported	Appropriate antibiotic treatment prescribing	
Standiford et al. (2012)	Hospital-wide	Patients admitted	Attending physicians	Appropriate antibiotic treatment prescribing	
Stenehjem et al. (2018)	Hospital-wide	Patients admitted	Prescribing physicians; pharmacists; nurses	Documenting; de-escalation: not clear, switching from intravenous to oral antibiotics; seeking clinical support; reviewing	Review: 48 h after antibiotic prescription. Seek clinical support between 8-5pm
Tang et al. (2019)	Medical wards	Patients admitted	Attending physicians	Appropriate antibiotic treatment prescribing; Reviewing antibiotics; documenting; de-escalation: broad to narrow, stop, switching from intravenous to oral antibiotics	
Tangden et al. (2011)	Hospital-wide	Patients admitted	Physicians	Appropriate antibiotic treatment prescribing	
Tedeschi et al. (2017)	Rehabilitation, Hospital-wide	Patients admitted	Physicians; nurses	Appropriate prescribing of both prophylactic and treatment antibiotics	
van Hees et al. (2008)	Internal medicine, gastroenterology, surgical ward, urology, pulmonary wards	Patients admitted	Physicians	Appropriate prescribing of both prophylactic and treatment antibiotics	
van Horrik et al. (2024)	Hospital-wide	Patients admitted with Asymptomatic bacteriuria	Prescribers	Appropriate antibiotic treatment prescribing; De-escalation	
Vercheval et al. (2016)	ICU, oncology, infectious diseases, haemotology, cardiology and respiratory wards	Patients admitted	Physicians; nurses	Documenting; seeking clinical support	
Wathne et al. (2018)	Gastroenterology, infectious diseases, pulmonary wards	Patients admitted	Resident physicians; resident consultants	Appropriate antibiotic treatment prescribing	
Willemsen et al. (2010)	Hospital-wide	Patients admitted	Attending physicians	De-escalation: switching from intravenous to oral antibiotics; appropriate antibiotic treatment prescribing	

We found that although most interventions targeted initial prescribing of antibiotics (*n* = 56, 96.6%), some interventions focused on other actions in the AMS behavioural pathway such as de-escalation of antibiotics (*n* = 13, 22.4%), and documentation of prescribing decisions (*n* = 9, 16.4%). The timeframe of the actions was reported in only 15 (25.8%) studies. The timeframe was mainly specified in the context of prescribing prophylactic antibiotics (*n* = 7; e.g. prescribe for up to 24 h post-surgery), prescribing treatment antibiotics (*n* = 3; e.g. 8-hour dosing intervals), de-escalation of antibiotics (*n* = 2; e.g. de-escalation after 48–72 h), reviewing antibiotics (*n* = 2; e.g. review at end of each month), and seeking clinical support (*n* = 1, anytime between 8:00–17:00). The actor of the behaviour targeted by interventions was well reported (*n* = 56, 96.6%), however the speciality of the doctors was not clearly reported. Physicians (i.e. doctors) were targeted most often as recipients of the interventions (35, 60.3%), although many studies lacked detail on the level of experience or grade of seniority and clinical specialty of the doctors targeted. Other healthcare provider roles forming the interdisciplinary team involved in AMS, such as pharmacists and nurses, were not frequently targeted by the included interventions. In terms of context, interventions were mostly implemented throughout the entire hospital (*n* = 27, 46.6%). Of those delivered for specific specialties/ wards, internal medicine (*n* = 14, 24.1%) and surgical wards (*n* = 10, 17.2%) were most frequently targeted. Most studies did not specify a target patient group (*n* = 40, 69.0%). In those that did, antibiotic prescribing for surgical patients was targeted most often (*n* = 7, 12.1%) followed by those with community acquired pneumonia (*n* = 3, 5.2%).

### Which modes of delivery have been used to deliver AMS education and training in hospital-based care?

Mode of delivery parameters, where reported, are described in Table 4. AMS education and training interventions were delivered using a range of modalities, most often via printed materials (*n* = 24, 41.4%), followed by face-to-face modality (*n* = 20, 34.5%), electronically (e.g., computerised decision support systems) (*n* = 12, 20.7%), via the internet (*n* = 10, 17.2%), telephone (*n* = 10, 17.2%), and email (*n* = 7, 12.1%). Hybrid interventions that consisted of face-to-face/ printed material and digital components (electronic, internet, telephone, or email modalities) were reported in 36 (62%) studies. The frequency of delivery for interventions was poorly reported, with missing data in 27 studies (46.5%). In the 12 studies that reported the duration of the intervention sessions, the average duration was 120 min, ranging from 10 to 480 min. For the 31 studies that did report how frequently intervention components were delivered, the most common frequency was daily (*n* = 9, 29%), followed by weekly (*n* = 6, 19.4%), and one-off delivery (*n* = 6, 19.4%). For studies reporting the provider, the AMS education and training interventions were most often delivered by a healthcare provider from a specialty more closely linked to AMS (e.g. infectious disease physician, AMS pharmacist) (*n* = 36, 62.1%).

**Table 4:** Modes of delivery for secondary care AMS education and training interventions in hospital-based care

Mode of delivery	*N* (%)	Examples
Face-to-face	20 (34.5)	*“…face-to-face structured educational interviews with Oncologists on the basis of specific antibiotic prescriptions to reinforce the principles of the correct use of antibiotics” (Molina* et al. 2019)
Printed material	24 (41.4)	*“The internal guideline was summarized on an educational pocket card and posters” (Popovski)*
Electronic	12 (20.7)	*“…electronic consultations were used mainly to adjust antibiotic doses according to therapeutic drug monitoring” (Tedeschi)*
Web	10 (17.2)	*“…physicians in the participating hospitals were invited to complete e-learning consisting of case-based questions about the community acquired pneumonia guidelines” (Schweitzer)*
Telephone	10 (17.2)	*“The [academic] detailers… made suggestions for alternative regimens… The encounter took place over the telephone…” (Solomon)*
Email	7 (12.1)	*“Guidelines and exclusion criteria were disseminated weekly via standardized emails to the surgical faculty as reminders” (Kashtan)*
Not reported	15 (25.9)	
Frequency		
One-off	6 (10.3)	*“First*,* clinical education was provided to the paediatric surgeons and surgical advanced practice providers on April 19*,* 2017…” (Seddik)*
Yearly	2 (3.5)	*“…yearly educational lectures for residents and paediatricians” (Barbieri)*
Twice a year	1 (1.7)	*“A total number of… 10 clinical sessions (1–2 per year)… were delivered after the initiation of the antimicrobial stewardship program …” (Molina* et al. 2019)
Every 3 months	1 (1.7)	*“54 clinical lessons were given*,* with a mean interval of 90 days (standard deviation = 43)” (Schweitzer)*
Monthly	5 (9.6)	*“15-minute educational sessions were conducted for the residents with a new session during morning rounds held every block*,* on a 4-week cycle”. (Irfan)*
Twice a month	(3.5)	*“An investigator presented teaching sessions to physicians in the clinician education firm twice during each 4-week rotation”. (Schwartz* et al. 2017)
Weekly	6 (10.3)	*“Weekly joint ward rounds were undertaken throughout the year with an infectious disease physician*,* a pharmacist*,* and junior ward-based members of the plastic surgery department*,* with individual patient review and ongoing educational reinforcement of guidelines.” (Daly)*
Twice a week	2 (3.5)	*“…antimicrobial stewardship rounds two afternoons weekly with an ID–trained clinical pharmacist”. (Tang)*
Three times a week	1 (1.7)	*“Three times a week*,* an infectious diseases pharmacist would discuss the antimicrobial prescriptions with the infectious diseases physician and join ward rounds with the physician”. (Huang)*
Daily	9 (15.5)	*“…an initial 3-month ‘intensive phase’… During the first phase*,* a dedicated ID specialist took part in the daily activities of the ward” (Carrara)*
Not reported	27 (46.6)	
Source		
Infectious diseases physician	18 (31.0)	*“…on-site education sessions were provided on different wards by infectious diseases physicians” (Corcione)*
Pharmacist	13 (22.4)	*“Pharmacist support of the initiative via ongoing education on the wards” (Gardiner*,* 2018)*
Microbiologist	9 (15.5)	*“…antibiotic prescribing advice provided by a senior microbiologist”. (Liebowitz)*
General pharmacist	9 (15.5)	*“Educational outreach visits: Oral communication by a clinical pharmacist highlighting the intervention during educational materials distribution” (Vercheval)*
Study staff/ project coordinator	10 (17.2)	*“The project manager coordinated all activities of coworkers involved in the project” (Willemsen)*
Antimicrobial Management Team	6 (10.3)	*“The Antimicrobial Utilisation Team provided structured feedback to prescribing physicians on the appropriateness of antimicrobial use” (Camins)*
Infectious diseases pharmacist	4 (6.9)	*“For all orders*,* the pharmacist either completes a vancomycin data form or notes the indication on the physician’s order. The vancomycin data form contains the guidelines for vancomycin use and functions as a data collection tool for the infectious diseases pharmacist*,* allowing him to provide feedback to pharmacists on orders that do not comply with the restriction guidelines.” (Belliveau)*
Infectious diseases specialist	4 (6.9)	*“…reassessment sessions of current antibiotic prescriptions by a single practitioner with the antibiotic referent and/or the infectious diseases specialist” (Roger)*
Infectious diseases consultant	4 (6.9)	*“The infectious diseases consultant visited patients*,* reviewed their clinical data*,* discussed the prescribed antibiotic treatment and diagnosis of the infectious syndrome with their physicians”. (Tedeschi)*
Pharmacy assistant	2 (3.5)	*“The ward’s pharmacy assistants were instructed to increase the physician’s awareness of the new regimens*,* and to ensure that the guidelines were followed”. (Knudsen)*
Nurse	2 (3.5)	*“…two senior emergency department clinicians… and a nurse… spent one on one time educating colleagues (doctors and pharmacists) about antibiotic prescribing recommendations”. (Buising)*
Senior clinician	1 (1.7)	
Senior departmental leaders	1 (1.7)	*“As a result of the educational material presented to senior departmental leaders*,* the department of obstetrics and gynaecology put in place several subsequent educational interventions aimed at bringing practice in line with our recommendations”. (Everitt)*
Quality improvement officer	2 (3.5)	*“Each hospital installed a Local Organising Committee*,* in which both contact persons participated and depending on the hospital… a quality improvement officer and/ or a nurse from the departments” (Spoorenberg)*
Not reported	8 (13.8)	

### Which intervention types and behaviour change techniques make up the content of AMS education and training in hospital-based care?

The summary of intervention types and behaviour change techniques in the studies are listed in Tables 5 and 6. Education was identified as an intervention type in almost all included interventions (*n* = 62, The next most frequent intervention types were Enablement (*n* = 58) and Persuasion (*n* = 57) identified in intervention arms.

**Table 5:** Identified behaviour change techniques in intervention and comparator arms

Behaviour change technique	Definition^a^ (Michie et al. 2013)	Frequency *N* (%)		Examples	
		Intervention *N* = 67	Comparator *N* = 25	Intervention	Comparator
1.1 Goal setting (behaviour)	*Set or agree on a goal defined in terms of the behaviour to be achieved*	20 (29.9)	1 (4.0)	*Setting an individual objective for behaviour change to increase appropriateness of their own antimicrobial prescribing. (McLellan)*	*Our goal: To ensure optimal antibiotic use to improve your patient’s outcomes and safety. (Stenehjem)*
1.2 Problem solving	*Analyse*,* or prompt the person to analyse*,* factors influencing the behaviour and generate or select strategies that include overcoming barriers and/or increasing facilitators*	5 (7.5)	0 (0.0)	*At these meetings… specific problems and improvement strategies discussed. (Tangden)*	
1.4 Action planning	*Prompt detailed planning of performance of the behaviour (must include at least one of context*,* frequency*,* duration and intensity).*	19 (28.4)	3 (12.0)	…*a plan with tailored improvement activites was sent. (Spoorenberg)*	*For this intervention*,* we created a new focused report to enable additional review of inpatients who were receiving > 1 antibiotic with anaerobic activity. Recommendations about redundant anaerobic regimens were made directly to the clinical teams. (Flett)*
1.5 Review behaviour goal(s)	*Review behaviour goal(s) jointly with the person and consider modifying goal(s) or behaviour change strategy in light of achievement. This may lead to re-setting the same goal*,* a small change in that goal or setting a new goal instead of (or in addition to) the first*,* or no change*	5 (7.5)	0 (0.0)	*These [prescribing] protocols… can evolve with time*,* being revisited and validated on a yearly basis. (Saizy-Callaert)*	
1.6 Discrepancy between current behaviour and goal	*Draw attention to discrepancies between a person’s current behaviour (in terms of the form*,* frequency*,* duration*,* or intensity of that behaviour) and the person’s previously set outcome goals*,* behavioural goals or action plans (goes beyond self-monitoring of behaviour)*	27 (40.3)	2 (8.0)	*The responsible physicians were contacted by a member of the local antimicrobial stewardship team… if the treatment they had initiated was not consistent with the guideline recommendation. (Schweitzer)*	*Three times a week*,* an infectious diseases pharmacist would discuss the antimicrobial prescriptions with the ID physician and join ward rounds with the physician… Prescribers who followed major inappropriate principles in antimicrobial use were asked to join the discussion. (Chang)*
1.8 Behavioural contract	*Create a written specification of the behaviour to be performed*,* agreed on by the person*,* and witnessed by another*	4 (6.0)	0 (0.0)	*Incorporation of the objectives of the ASP to the annual agreement signed by the department (Molina*,* 2019)*	
1.9 Commitment	*Ask the person to affirm or reaffirm statements indicating commitment to change the behaviour*	2 (3.0)	0 (0.0)	*Stating a numerical ‘commitment to change’ between 1 and 10 (McLellan)*	
2.1 Monitoring of behaviour by others without feedback	*Observe or record behaviour with the person’s knowledge as part of a behaviour change strategy*	4 (6.0)	0 (0.0)	*The interns and residents were not aware that their ordering patterns were being studied. (Solomon)*	
2.2 Feedback on behaviour	*Monitor and provide informative or evaluative feedback on performance of the behaviour (e.g. form*,* frequency*,* duration*,* intensity)*	35 (52.2)	4 (16.0)	…*Sessions were adversarial discussions with the prescribers*,* regarding their own patients with their own prescriptions*,* so that they could best appreciate the merits of the STGs [guidelines]. (Roger)*	…*we created a new focused report to enable additional review of inpatients who were receiving > 1 antibiotic with anaerobic activity. Recommendations about redundant anaerobic regimens were made directly to the clinical teams. (Flett)*
2.3 Self-monitoring behaviour	*Establish a method for the person to monitor and record their behaviour(s) as part of a behaviour change strategy*	4 (6.0)	1 (4.0)	*Our stewardship team (Drs. Lee and Frenette) developed an online checklist that formalized each time-out audit into a step-by-step process meant to approximate how an infectious diseases specialist might approach prospective audit and feedback…. On each audit day*,* the senior resident would apply the checklist to all patients receiving antibiotics. (Lee)*	*Bundle components included (1) an antimicrobial rationale checklist for daily use on attending rounds*,* (2) a templated progress note integrated into the electronic medical record (EMR) to document key elements of antimicrobial use. (Tang)*
2.7 Feedback on outcome(s) of behaviour	*Monitor and provide feedback on the outcome of performance of the behaviour*	8 (11.9)	1 (4.0)	*The clinician-educators also distributed graphs and summaries of resistance patterns in our institution (Solomon)*	*An infectious diseases physician reviewed prespecified positive cultures (e.g.*,* all positive blood cultures*,* cultures with highly resistant Enterobacteraciae) Monday through Friday and contacted providers with recommendations. (Stenehjem)*
3.1 Social support (unspecified)	*Advise on*,* arrange or provide social support (e.g. from friends*,* relatives*,* colleagues*,*’ buddies’ or staff) or non-contingent praise or reward for performance of the behaviour.*	1 (1.5)	0 (0.0)	*[Local opinion leaders] were asked to encourage guideline-adherent treatment throughout the intervention period (e.g.*,* during handover meetings). (Schweitzer)*	
3.2 Social support (practical)	*Advise on*,* arrange*,* or provide practical help (e.g. from friends*,* relatives*,* colleagues*,* ‘buddies’ or staff) for performance of the behaviour*	34 (50.7)	3 (12.0)	*Clinicians at all participating hospitals had access to an ID telephone hotline*,* staffed by an attending ID physician 24 h a day*,* 7 days a week to answer clinical questions (Stenehjem)*	*Three times a week*,* an infectious diseases pharmacist would discuss the antimicrobial prescriptions with the ID physician and join ward rounds with the physician. (Chang)*
4.1 Instruction on how to perform the behaviour	*Advise or agree on how to perform the behaviour (includes ‘Skills training’)*	56 (83.6)	7 (28.0)	*Lecture series on clinical infectious diseases*,* focusing on the appropriate use of antibacterial drugs (Kishida)*	…*access to hospital-wide antimicrobial stewardship programs*,* including institutional infection management guidelines implemented in 2004 to support the diagnosis and treatment of common infection syndromes… (Schwartz* et al. 2017)
4.2 Information about antecedents	*Provide information about antecedents (e.g. social and environmental situations and events*,* emotions*,* cognitions) that reliably predict performance of the behaviour*	10 (14.9)	0 (0.0)	*When diagnostic or laboratory data indicated the appropriateness of initiating an alternative therapeutic regimen (cefazolin or cefuroxime)*,* the* *clinical pharmacist communicated these findings to the prescribing physician. (Skaer)*	
5.1 Information about health consequences	*Provide information (e.g. written*,* verbal*,* visual) about health consequences of performing the behaviour*	34 (50.7)	1 (4.0)	…*emphasizing changing susceptibility patterns and syndromes associated with antimicrobial overuse. (Schwartz*et al. 2007)	[Supplementary material] *Why should I perform IV to PO conversions? Reason 1: It has many benefits for the patient and the hospital… Reduced exposure to nosocomial pathogens through the IV site 1*,* Decreased risk of phlebitis. (Stenehjem)*
5.3 Information about social and environmental consequences	*Provide information (e.g. written*,* verbal*,* visual) about social and environmental consequences of performing the behaviour*	7 (10.4)	1 (4.0)	*A common presentation template was prepared for all intervention sessions with information about… local antibiotic sales statistics (Wathne)*	[Supplementary material] *Why should I perform IV to PO conversions? Reason 1: It has many benefits for the patient and the hospital… Lower costs (drug cost*,* IV tubing*,* syringes*,* IV pumps). (Stenehjem)*
6.1 Demonstration of the behaviour	*Provide an observable sample of the performance of the behaviour*,* directly in person or indirectly*	10 (14.9)	1 (4.0)	*The attendees were presented with cases of appropriate and inappropriate use of both antibiotics (Chang)*	[Supplementary material] Demonstrates how to *review the type*,* source*,* and status of the culture. (Stenehjem)*
6.2 Social comparison	*Draw attention to others’ performance to allow comparison with the person’s own performance*	5 (7.5)	1 (4.0)	*Each participant received data about their own antimicrobial prescribing (both appropriate and suboptimal) and*,* for comparison*,* collated information about antimicrobial prescribing in the whole intervention group. (McLellan)*	*A feedback report was sent by mail to the contact persons summarizing the results of the baseline measurement*,* i.e. own department’s quality improvement performance scores and scores of the whole multi-faceted strategy group. (Spoorenberg)*
7.1 Prompts/cues	*Introduce or define environmental or social stimulus with the purpose of prompting or cueing the behaviour. The prompt or cue would normally occur at the time or place of performance*	31 (46.3)	2 (8.0)	*Guidelines and exclusion criteria were disseminated weekly via standardized emails to the surgical faculty as reminders. (Kashtan)*	*A templated progress note integrated into the electronic medical record (EMR) to document key elements of antimicrobial use. (Tang)*
8.2 Behavioural substitution	*Prompt substitution of the unwanted behaviour with a wanted or neutral behaviour*	20 (29.9)	2 (8.0)	*Implementation of the use of oral fosfomycin*,* nitrofurantoin*,* or cotrimoxazole instead of fluoroquinolones and third-generation cephalosporins for empiric treatment of non-severe urinary tract infections. (Tedeschi)*	*Levofloxacin was considered for respiratory infections only*,* and more specifically as an* *alternative for patients allergic to penicillin or in case of severe Legionella pneumophila infection. (Roger)*
9.1 Credible source	*Present verbal or visual communication from a credible source in favour of or against the behaviour*	40 (59.7)	3 (12.0)	*An infectious diseases physician communicated the content and rationale for the new recommendations. (Tangden)*	*The third team received the educational bundle in addition to antimicrobial stewardship from an internal medicine–trained clinical pharmacist embedded into the team during daily weekday morning attending rounds. (Tang)*
10.2 Material reward (behaviour)	*Arrange for the delivery of money*,* vouchers or other valued objects if and only if there has been effort and/or progress in performing the behaviour*	1 (1.5)	0 (0.0)	*To engage the participants and improve response rates*,* we awarded a $10*,* $15*,* or $25 gift card to the 3 participants with the highest final scores. (Flett)*	
10.3 Non-specific reward	*Arrange delivery of a reward if and only if there has been effort and/or progress in performing the behaviour*	1 (1.5)	0 (0.0)	*The performance score would be increased 1 point according to the growth of prescription of unrestricted antimicrobials. The performance score was associated with bonus. (Liu)*	
12.1 Restructuring the physical environment	*Change*,* or advise to change the physical environment in order to facilitate performance of) the wanted behaviour or create barriers to the unwanted behaviour (other than prompts/cues*,* rewards and punishments*	33 (49.3)	3 (12.0)	*Physicians were informed of the restriction program through announcements in the medical staff newsletter*,* signs posted on all the nursing units*,* a message on the hospital computer… (Belliveau)*	*Posters of institutional antibiotic guidelines* *for common disease syndromes posted in team workrooms. (Tang)*
12.2 Restructuring the social environment	*Change*,* or advise to change the social environment in order to facilitate performance of the wanted behaviour or create barriers to the unwanted behaviour (other than prompts/cues*,* rewards and punishments)*	28 (41.8)	4 (16.0)	*The prescription was validated on the next working day by a member of the antimicrobial stewardship tem (Garcia-Martinez)*	*A study Infectious Diseases pharmacist reviewed all requests for restricted antibiotics*,* and an infectious diseases physician reviewed prespecified positive cultures. (Stenehjem)*
12.5 Adding objects to the environment	*Add objects to the environment in order to facilitate performance of the behaviour*	36 (53.7)	4 (16.0)	*Buttons promoting “Save the Quinolones” were also distributed to hospital staff and worn to increase visibility. (Smoke)*	*All medical teams*,* regardless of randomization allocation*,* received at the start of each month pocket-sized cards that contained the Grady Memorial Hospital guidelines for use of antimicrobial agents*,* including guidelines for the use of the targeted study drugs: piperacillin-tazobactam*,* vancomycin*,* and levofloxacin. (Camins)*
13.2 Framing	*Suggest the deliberate adoption of a perspective or new perspective on behaviour (e.g. its purpose) in order to change cognitions or emotions about performing the behaviour*	1 (1.5)	0 (0.0)	*Antibiotics were divided into three catalogues with different levels of restrictions (unrestricted antimicrobials*,* restricted antimicrobials*,* and special antimicrobials) according to antimicrobial resistance*,* safety*,* effectiveness*,* and price. (Liu)*	
14.2 Punishment	*Arrange for aversive consequence contingent on the performance of the unwanted behaviour*	1 (1.5)	0 (0.0)	*The performance score would be deducted* *0.5–2 points depending on the increase of antibiotic consumption compared with prior month (Liu)*	

**Table 6:** Summary of intervention types as defined by the behaviour change wheel (Michie et al. 2011)

Intervention type	Definition (Michie et al. 2011)	Frequency *N* (%)		Example	
		Intervention *N* = 67	Comparator *N* = 25	Intervention	Comparator
Enablement	*Increasing means/ reducing barriers to increase capability (beyond education or training) or opportunity (beyond environmental restructuring)*	58 (86.6)	6 (24.0)	*Monthly educational meetings with the counselor team to reinforce the homogeneity of criteria and to encourage continuous learning regarding the main aspects of antibiotic use. (Molina* et al. 2017)	*For this intervention*,* we created a new focused report to enable additional review of inpatients who were receiving > 1 antibiotic with anaerobic activity. (Flett)*
Education	*Increasing knowledge or understanding*	62 (92.5)	7 (28.0)	*A dedicated ID specialist took part in the daily activities of the ward to observe common practices*,* discuss antibiotic prescriptions*,* and increase awareness and knowledge regarding AMR and hospital-acquired infections. (Carrara)*	*All residents*,* interns*,* and medical students received an educational intervention bundle at the start of their rotation designed to promote prescriber-initiated critical thinking about antimicrobial management. (Tang)*
Training	*Imparting skills*	33 (49.3)	1 (4.0)	*The feedback workshops were designed to increase participants’ ability to prescribe appropriately by addressing knowledge gaps*,* discussing social and behavioural aspects of prescribing*,* and encouraging reflection. (McLellan)*	The supplementary material shows how to perform AMS skills (reviewing, de-escalation, seeking clinical support, evaluating for “bug-drug mismatches”). *(Stenehjem)*
Modelling	*Providing an example for people to aspire to or imitate*	10 (15)	1 (4.0)	*Academic detailing sessions focused on recently admitted infectious diseases patients*,* including cases with treatment both adherent and non-adherent to guidelines. (Wathne)*	The supplementary material includes instructions and images to model/ demonstrate how to perform reviews by completing an antibiotic timeout form. *(Stenehjem)*
Restructuring the environment	*Changing the physical or social context*	52 (77.6)	5 (20.0)	*Distribution of posters in every ward (Vercheval)*	*During stewardship rounds*,* discussions between the internal medicine-trained clinical pharmacist and primary team continued until consensus was reached regarding each patient’s antibiotic plan. (Tang)*
Incentivization	*Creating an expectation of reward*	4 (6.0)	0 (0.0)	*To engage the participants and improve response rates*,* we awarded a $10*,* $15*,* or $25 gift card to the 3 participants with the highest final scores. (Flett)*	
Coercion	*Creating an expectation of punishment or cost*	1 (1.5)	0 (0.0)	*The clinical department would be fined for failure to achieve the goals. (Liu)*	
Persuasion	*Using communication to induce positive or negative feelings or stimulate action*	57 (85.1)	7 (28.0)	*The detailers provided each ordering physician with a copy of the guidelines and made suggestions for alternative regimens based on these recommendations*,* but the final drug choice was always left to the interns and residents. (Solomon)*	*Infectious diseases pharmacists reviewed the appropriateness*,* dosage*,* frequency*,* and treatment duration of prescribers’ cases and reported to the online computerized antibiotic control system. The prescribers would be telephone-called if an infectious diseases pharmacist identified inappropriate antimicrobial use. (Chang)*
Restriction	*Using rules to reduce the opportunity to engage in the target behaviour (or increase the target behaviour by reducing the opportunity to engage in competing behaviours)*	8 (11.9)	1 (4.0)	*A named-patient prescription form is provided for costly antibiotics and those known to have a marked impact on bacterial ecology. These drugs can only be prescribed by a senior hospital physician. (Saizy-Callaert)*	*Program 2 and 3 hospitals also implemented antibiotic restrictions. The following antibiotics were restricted: daptomycin*,* linezolid*,* imipenem…(Stenehjem)*

We identified 29/93 (31%) possible BCTs from the BCTTv1 Taxonomy across the interventions. Each AMS education and training intervention included on average 8 BCTs (range: 1–16), see Table 5. The most frequent BCTs were ‘Instruction on how to perform the behaviour’ (n = 56, 83.6%), ‘Credible source’ (n = 40, 59.7%), and ‘Feedback on behaviour’ (n = 35, 52.2%). The comparator arms included on average 3 BCTs (range: 0–16), with the four most common BCTs being ‘Instruction on how to perform the behaviour’ (n = 7, 28%), ‘Feedback on Behaviour’ (n = 4, 16%), ‘Restructuring the social environment’ (n = 4, 16%) and ‘ Adding objects to the environment’ (*n* = 4, 16%).

### Risk of bias

The risk of bias was assessed for the 27 studies that measured the primary outcome (antibiotic consumption) following the Cochrane guidance (Boutron 2023) RoB2 and ROBINS-I. 14 (52%) were assessed as having a low risk of bias, 1 (3%) as moderate risk, 1 (3%) as serious risk, and 11 (43%) as critical risk of bias. The judgements for each domain within studies are displayed in Supplementary file 4.

### How do Intervention effects vary according to targeted behaviours, mode of delivery parameters, component BCTs and BCW intervention types?

Our original protocol outlined plans for a meta-regression which was conducted on 26 studies prior to the updated search. However, we were unable to update the meta-analysis with the data from the additional one study identified in the updated search due to lack of capacity within the team after the funding period.

The effectiveness analyses included 26 interrupted time-series studies measuring antibiotic consumption. Analyses for other outcome measures and study designs were not performed due to insufficient data.

Meta-regression analyses assessing the impact of target action and context variables on antibiotic consumption were inconclusive (*p* values = 0.254, 0.91, 0.30, respectively). Therefore, we found no evidence that intervention effectiveness was influenced by whether interventions targeted de-escalation vs. starting an antibiotic (action), or whether implemented in multiple or single wards (context).

The meta-regression for mode of delivery including all covariates showed that studies using face-to-face modality led to greater reductions in antibiotic consumption compared to those that did not (β= − 3.42, 95% CI: − 6.55 to − 0.30, k = 20). Although after backward elimination this association was no longer statistically significant (*p* = 0.08). Sensitivity analyses conducted using a minimum of 5 studies per covariate category detected no moderator effect (k = 18). The sensitivity analysis with outliers removed also did not identify any moderator effects. There was no evidence that the source/provider of the intervention (AMS specific vs. general HCP) influenced intervention effectiveness (*p* = 0.30).

The meta-regression for BCTs did not detect any effect of individual component BCTs on antibiotic consumption, and the meta-CART did not detect any interaction effects. Sensitivity analyses also did not reveal any effects of BCTs on antibiotic consumption. The meta-regression analysis for intervention types were also inconclusive (k = 27). However, the sensitivity analysis with outliers removed (k = 20) identified significant effects of modelling (β= − 2.23 (95% CI: − 427 to − 0.18), and restriction intervention types (β = 2.95 (95% CI: 1.10 to 4.79). Modelling is defined as providing an example for people to aspire to imitate and restriction is defined as using rules to reduce the opportunity to engage in the target behaviour (or to increase the target behaviour by reducing the opportunity to engage in competing behaviours) (Michie et al. 2011). Examples are set out below and in Table 6.

- MODELLING example: Academic detailing sessions focused on recently admitted infectious diseases patients, including cases with treatment both adherent and non-adherent to guidelines. (Wathne)
- RESTRICTION example: A named-patient prescription form is provided for costly antibiotics and those known to have a marked impact on bacterial ecology. These drugs can only be prescribed by a senior hospital physician. (Saizy-Callaert)

The meta-CART analysis for intervention types (k = 27) and the sensitivity analysis with outliers removed (k = 20) detected no statistically significant associations.

### Cost-effectiveness

The cost estimates for the intervention in each study and the cost scenario analysis results are presented in full in Supplementary file 3. There is considerable variation in cost estimates with a few interventions associated with very high-cost values. The study by Kjaersgaard et al., in 2019 has the highest cost, with the cost of £1,046,316 and the study by Belliveau in 1996 had the lowest cost of £8,323. Most scenario analyses had little effect on the relative cost estimates across studies.

Characteristics associated with greater effectiveness and lower cost (South-East quadrant on the plane) may be more likely to be associated with cost-effective interventions. The strength of evidence is depicted by the colour of the letter label. ‘< 0.2, < 0.1’ indicates that the p-value for the effect results was < 0.2 and the *p*-value for the cost results was < 0.1.

The only factor that had a *p*-value for effect and cost < 0.1 was face-to-face mode of delivery.

## Discussion

The aim of this review was to describe and analyse the behavioural content and delivery of AMS education and training interventions in hospital-based care as a basis for identifying opportunities to inform the design of future, or refinement of existing, AMS education and training interventions in research and practice. We specified which and whose behaviours were targeted, synthesised the behaviour change content in interventions, and extracted information on the intervention characteristics including the modes of delivery. We also assessed the effectiveness of the AMS education and training interventions in relation to different components within the interventions and assessed their cost-effectiveness.

We identified that AMS education and training is more effective when delivered in person (face-to-face). Although delivering and attending in person training can often be perceived as more burdensome, time and resource consuming than digital modes of delivery, in our analysis face-to-face modality was the only factor found to be cost-effective. Though existing evidence is limited, particularly in relation to AMS, this finding is novel given previous evidence of delivering education to healthcare professionals more broadly suggests no difference in effectiveness between the delivery modalities and highlights the likely preference for online training to increase reach (Richmond et al. 2017). A review by McGhee et al., 2024 found digital teaching of clinical skills demonstrated improved or comparable outcomes to in-person teaching in medical students. However, specific modes of delivery of the teaching were most effective in improving skills and knowledge, but more specific skills such as cardiac auscultation varied (McGee et al. 2024). A balance may be a hybrid model which has become a common delivery approach within higher education settings and healthcare settings post COVID but may require some further disentangling of the most appropriate content for online and face-to-face delivery.

AMS education and training was also found to be more effective when it incorporates the behaviour change strategies of modelling and restriction. This aligns with the finding of the original Cochrane Review of AMS interventions in secondary care (Davey et al. 2013), which identified that AMS interventions more broadly (beyond education and training) that included restriction were independently associated with increased compliance with antibiotic policies. Restriction (i.e. restricting access to certain antimicrobials) operates through reducing physical opportunity to prescribe antimicrobials and could therefore arguably be seen as more of a co-intervention strategy to be delivered alongside education and training. Modelling, however, could be delivered as part of or alongside education and training (e.g. via role plays, demonstrations). The role of vicarious learning, or modelling, has been strongly emphasised in Social Learning Theory (Bandura and Walters 1977), which acknowledges that most human behaviour is learned observationally through modelling.

Consistent with findings from previous reviews of AMS interventions undertaken in hospital-based care (Talkhan et al., 2020), we found that interventions lacked a theoretical basis, with only 7 (12.7%) studies reporting the use of behavioural theory to inform intervention design. Whilst some studies did report carrying out some prior formative work to inform their interventions (*n* = 11, 19.0%) such as literature reviews or understanding barriers and enablers to specified behaviours, this does not to appear to be the norm. Research suggests the importance of developing interventions based on theory (Davey et al. 2013; Hagger and Weed 2019; Michie et al. 2011) as theories explain why people behave the way they do and can help us to develop interventions that target the correct behavioural influences. Carrying out formative work and reporting this in such studies is a step in the right direction, as this can help tailor interventions to overcome identified barriers. However, behavioural science and theory plays an essential role in optimising AMS education and training but often there is a gap in application due to lack of expertise, confidence to apply and understanding (Sirota et al. 2024; Turner et al. 2023).

Almost half of included studies (*n* = 26, 47.3%) failed to describe the frequency of the intervention. This aligns with previous work which identified that reporting of intervention components is substandard (Dijkers 2021) and suggests a need for improved reporting of the active content of interventions. Whilst this suggests that future studies should adhere to reporting guidelines (e.g. the TIDieR checklist, CONSORT guidelines) to facilitate evidence syntheses to produce more definitive conclusions, increased uptake may require further development or tailoring of such frameworks (Dijkers 2021). As well as better reporting of intervention components, our analysis also suggests a need to use behavioural reporting frameworks such as AACTT (Presseau et al. 2019) to better define and describe the targeted AMS related behaviours.

### Limitations

A key limitation is our deviation from protocol in relation to the meta-analysis. Our original protocol outlined plans for a meta-regression, which was conducted on 26 studies prior to the updated search. However, we were unable to update the analysis with the data from the additional one study identified in the updated search due to lack of capacity and skills within the team after the funding period. Whilst it is unlikely that the one additional study would have a significant impact on these findings, we cannot be certain of this.

Our search strategy required education and/ or training terms to be used in the title or abstract which meant relevant studies without these terms were excluded. However, systematic review methodology advises a calibration of criteria that balances sensitivity with specificity, and so the omission of relevant studies in the selection process is to be expected when using a search strategy containing focused criteria. Other limitations originate from the primary studies which may limit the generalisability of our findings, for example most studies were from high-income countries, including five from the Netherlands where rates of prescribing and resistant pathogens are low. The heterogeneity of the outcome measures meant there was sufficient data for only antibiotic consumption, whereas measures of appropriateness and resistance rates are deemed more useful in this context. Finally, information regarding intervention characteristics was missing from many of the primary studies which reduces the accuracy of our estimates.

### Implications for research and practice

In most studies education and training was a co-intervention used alongside other strategies (e.g. audit and feedback) rather than a stand-alone intervention, which makes it difficult to isolate its impact (Satterfield et al., 2020). However, synthesising evidence from studies of educational interventions that closely resemble real-world practice enhances the external validity of our findings and is consistent with Centre for Disease Control (CDC) guidelines that recommend combining education with other strategies in AMS interventions (CDC, 2021).

Improvements are needed in standardising outcome measures to enable meta-analyses. In practice, policy and research, different outcomes are used, based on different guidelines, relevance and importance. For example, DDDs (or equivalent) may not possibly resonate with the health workforce, as this is commonly a terminology used with national policymakers. Moreover, there is scope for implementation outcomes to enhance the evaluation of the implementation of interventions (Lengnick-Hall et al., 2022). Future interventions should expand on the range of implementation outcomes used, particularly those important to AMS such as sustainability of the intervention and its effectiveness. Authors may wish to refer to Proctor et al. (2011) or other relevant implementation outcome definitions, such as those described by the NIHR. Such outcome frameworks differ by how similar measures are defined, for example, Proctor et al. (2011) describes adoption to be separate to fidelity while the NIHR framework instead defines adoption as a fidelity construct known as ‘treatment enactment’.

Our results suggest a few key recommendations for those designing and delivering AMS education and training. Firstly, given AMS is behaviourally complex, we, along with others in the field, strongly advocate using behavioural science in the design of interventions to optimise their impact (Schweitzer et al., 2020). For example, interventions identified in this review targeted a narrow range of behaviours and actors involved in AMS. It is important to be more precise and specific in whose and which behaviours the intervention targets, given we know different behaviours and actors are driven by different influences. Secondly, at least some elements should be delivered face-to-face, and thirdly, interventions should aim to include restriction (removing or constraining choices) and modelling (credible role models performing the desired behaviour) elements within their design. Whilst we identified several BCTs within the interventions, our analysis did not identify effects of any individual or combination of these. We recommend the use of other evidence-based resources and guidance when identifying relevant BCTs, such as the Human Behaviour Change Project (https://www.humanbehaviourchange.org/) (Marques et al. 2023), alongside the new Behaviour Change Technique Ontology (https://www.bciontology.org/) (Marques et al. 2023).

## Conclusions

AMS education and training interventions delivered face-to-face, via printed materials, or incorporating restriction or modelling may be effective in reducing antimicrobial consumption. Modelling support social learning of the wider health workforce team by allowing peers to observe their colleagues performing the desired behaviours and is more likely to be feasible for future interventions to include. In comparison to the use of restrictive strategies as these require careful consideration due to potential risks, such as negative impacts on workplace culture and challenges in long-term sustainability. Further evidence from rigorously designed and behaviourally informed studies, with transparent reporting of intervention components, is needed to better determine the specific content and methods associated with effective AMS education and training.

## Supplementary Information

Below is the link to the electronic supplementary material.

Supplementary Material 1.

Supplementary Material 2.

Supplementary Material 3.

Supplementary Material 4.

## Data Availability

Data is available on request.

## Abbreviations

AACTT: Actor, Action, Context, Target and Time framework
AMR: Antimicrobial resistance
AMS: Antimicrobial stewardship
BCT: Behaviour change technique
BCTTv.1: Behaviour change technique taxonomy version 1
BCW: Behaviour change wheel
PRISMA: Preferred reporting items for systematic reviews and meta-analyses
