# Supplementary material for: A systematic review and meta-analysis to identify behavioural content and active ingredients of antimicrobial stewardship education and training interventions in hospital-based care settings
Source: Antimicrob Resist Infect Control. 2025 Dec 18;15:10. doi: 10.1186/s13756-025-01660-0 (PMC12829054; doi:10.1186/s13756-025-01660-0)
Supplement: Supplementary file 1 — Supplementary Material 1. [file 13756_2025_1660_MOESM1_ESM.docx]

**Supplementary file 1: Search strategy**

| **EMBASE** |
| --- |
| 1., exp antibiotic resistance/ |
| 2., AMR.ti,ab., |
| 3., ((antimicrobial or anti?microbial or antibacterial or anti?bacterial or antibiotic or bacteria* or drug*) and (resistant or resistance)).ti,ab. |
| 4., or/1-3 |
| 5., (education$ or continuing?education$ or cme or ‘continuing professional development’ or cpd or 'academic detailing' or training or train or teaching or initiative or workshop* or seminar* or module* or session* or curriculum or learn* or skill* or knowledge).ti,ab. |
| 6., (education$ and (outreach or visit? meeting? or seminar? or conference? or course? or material? or game? or intervention?)).ti,ab. |
| 7., 5 or 6 |
| 8., (management or overprescrib$ or overuse or overusing or pattern? or prescribing or prescri* or provision or prudent$ or stewardship or AMS or rational or unnecessary or use or usage or consumption or utili?ation).ti,ab. |
| 9., exp secondary health care/ |
| 10. exp hospital/ |
| 11., (hospital* or 'secondary care' or ' secondary setting' or 'emergency care' or 'emergency setting?' or 'emergency department?').ti,ab. |
| 12., or/9-11 |
| 13., 4 and 7 and 8 and 12 |
| 14., (randomi#ed controlled trial or controlled clinical trial).pt. or randomi#ed.ab. or placebo.ab. or clinical trials as topic.sh. or randomly.ab. or trial.ti. |
| 15., (pre?intervention? or preintervention? or pre intervention? or post?intervention? or postintervention? or post intervention?).ti,ab. |
| 16., (pre?post or pre test$ or pretest$ or posttest$ or post test$ or (pre adj5 post)).ti,ab. |
| 17., (pre?workshop or post?workshop or (before adj3 workshop) or (after adj3 workshop)).ti,ab. |
| 18., trial.ti. or ((study adj3 aim?) or our study).ab. |
| 19., (before adj10 (after or during)).ti,ab. |
| 20., (quasi?experiment$ or quasiexperiment$ or quasi random$ or quasirandom$ or quasi control$ or quasicontrol$ or ((quasi$ or experimental) adj3 (method$ or study or trial or design$))).ti,ab,hw. |
| 21., (time series adj2 interrupt$).ti,ab,hw. |
| 22., (time points adj3 (over or multiple or three or four or five or six or seven or eight or nine or ten or eleven or twelve or month$ or hour? or day? or "more than")).ab. |
| 23., (multi?centre or multi?center).ti,ab. |
| 24., or/14-23 |
| 25., “comment on".cm. or review.ti,pt. |
| 26., review.ti. |
| 27., (rat or rats or cow or cows or chicken? or horse or horses or mice or mouse or bovine or animal?).ti. |
| 28., exp animals/ not humans.sh. |
| 29., (animal$ not human$).sh,hw. |
| 30, or/25-29 |
| 31., 24 not 30 |
| 32., 13 and 31 |
| 33., limit 32 yr +’2015-2022’ |
| **MEDLINE** |
| 1., (AMR).ti,ab. |
| 2., *Drug Resistance, Microbial/ |
| 3., *Drug Resistance, Bacterial/ |
| 4., or/1-3 |
| 5., (antibiotic? or antimicrobial* or anti?microbial* or antibacterial* or anti?bacterial or bacteria*).ti,ab. |
| 6., (resistant or resistance).ti,ab. |
| 7., 5 and 6 |
| 8., 4 or 6 |
| 9., (management or overprescrib$ or overuse or overusing or prescri* or provision or prudent$ or stewardship or AMS or rational or unnecessary or use or usage or consumption or utili?ation).ti,ab. |
| 10., (education$ or continuing?education$ or cme or 'continuing professional development' or cpd or 'academic detailing' or training or train or teaching or workshop* or seminar* or module* or session* or curriculum or 'inter?professional education').ti,ab. |
| 11., (education$ and (outreach or visit? or meeting? or seminar? or conference? or course? or material? or game? or intervention?)).ti,ab. |
| 12., 10 or 11 |
| 14., exp secondary care/ |
| 15., exp hospitals/ |
| 16., (hospital* or 'secondary care' or ' secondary setting' or emergenc*).ti,ab. |
| 17., or/14-16 |
| 18., 8 and 9 and 12 and 17 |
| 19., (randomi#ed controlled trial or controlled clinical trial).ti,ab,pt. or randomi#ed.ab. or placebo.ab. or clinical trials as topic.sh. or randomly.ab. or trial.ti. |
| 20., (pre?intervention? or preintervention? or pre intervention? or post?intervention? or postintervention? or post intervention?).ti,ab. |
| 21., (pre?post or pre test$ or pretest$ or posttest$ or post test$ or (pre adj5 post)).ti,ab. |
| 22., (pre?workshop or post?workshop or (before adj3 workshop) or (after adj3 workshop)).ti,ab. |
| 23., (quasi?experiment$ or quasiexperiment$ or quasi random$ or quasirandom$ or quasi control$ or quasicontrol$ or ((quasi$ or experimental) adj3 (method$ or study or trial or design$))).ti,ab,hw. |
| 24., (time series adj2 interrupt$).ti,ab,hw. |
| 25., ("time points" adj3 (over or multiple or three or four or five or six or seven or eight or nine or ten or eleven or twelve or month$ or hour? or day? or "more than")).ab. |
| 26., (multi?centre or multi?center).ti,ab. |
| 27., or/19-26 |
| 28., "comment on".cm. or review.ti,pt. |
| 29., review.ti. |
| 30., (rat or rats or cow or cows or beef or chicken? or horse or horses or mice or mouse or bovine or animal?).ti. |
| 31., exp animals/ not humans.sh. |
| 32., (animal$ not human$).sh,hw. |
| 33., or/28-32 |
| 34., 27 not 33 |
| 36., 18 and 34 |
| 37., Limit 36 to yr + ‘2010–2022’ |
| **CENTRAL** |
| Antimicrobial Stewardship in Title Abstract Keyword |
| AND |
| (antibiotic? or antimicrobial* or anti?microbial* or antibacterial* or anti?bacterial or bacteria* or AMS) in Title Abstract Keyword |
| AND |
| (management or overprescrib$ or overuse or overusing or prescri* or provision or prudent$ or stewardship? or rational or unnecessary or 'use' or usage or consumption or utili?ation) in Title Abstract Keyword |
| AND |
| (education$ or continuing?education$ or cme or 'continuing professional development' or cpd or 'academic detailing' or training or train or teaching or workshop* or seminar* or module* or session* or curriculum or 'inter?professional education) in Title Abstract Keyword |
| AND |
| (hospital* or 'secondary care' or 'secondary setting' or 'emergency care' or 'emergency setting*' or 'emergency department*') in Title Abstract Keyword |
