# Supplementary material for: A systematic review and meta-analysis to identify behavioural content and active ingredients of antimicrobial stewardship education and training interventions in hospital-based care settings
Source: Antimicrob Resist Infect Control. 2025 Dec 18;15:10. doi: 10.1186/s13756-025-01660-0 (PMC12829054; doi:10.1186/s13756-025-01660-0)
Supplement: Supplementary file 2 — Supplementary Material 2. [file 13756_2025_1660_MOESM2_ESM.docx]

# Health Economics Objectives and Methods

## Objectives

The economics objectives were to:

- Cost the education and training interventions in the studies included in the review
- Evaluate the average costs associated with the education and training behaviour change characteristics
- Identify if there is evidence that any of these behaviour change characteristics may be more likely to be part of cost-effective interventions than others

The education and training behaviour change characteristics assessed were

- Behaviour change techniques
- Behaviour change wheel items
- Modes of delivery
- Context items

These have all been described in the main report section summarising Work package 1 (p.12-17). Behaviour change characteristics of education and training interventions are descriptors of components of an intervention. This analysis evaluates whether these components may be associated with cost-effective interventions in order to help inform the design of education and training interventions.

## Methods

The interventions investigated in the studies included in the systematic review (see description of WP1 p. 12-17) were costed. The cost of the intervention was the cost of delivering it over the duration of the intervention in the study. No cost outcomes were included in the analysis. The measures of benefit were the relative effect measures evaluated in the effectiveness section (WP1 p16-17).

### 1 Resource use

Resource items for data extraction for the included studies were developed by HS and SR. These were tested by SG and KC. Due to poor reporting of intervention characteristics, resource use questions specific to individual studies were developed and these were sent to authors. Responses were received from 12 authors. Questions were also sent to a clinical expert on typical resource use to help guide resource use assumptions where information gaps remained (see Table C1). HS and SR checked the resource use data extracted with the author responses and the study papers to complete the resource use data set.

*Table C1: Responses from clinical expert on reasonable resource use*

| **List of Questions** |
| --- |
| 1. If you were producing a 2-day CME-accredited training event for consultants and junior doctors,  - How many staff would you employ to produce the event? **3-5** - What experience would you employ (e.g. AMS consultant, pharmacist, clinical lecturer, other)? **Microbiology consultant, AMS pharmacist, clinical research fellow** - How many days would you plan for each person to spend developing it? **2-4 days preparation time in total so half to one day per person** - How many people would you employ to deliver the event? Depends on scale – admin + people doing the training sessions + facilitators for breakout group e.g. 10 people - Where would you hold such an event? **Again depends on scale. Local hospital – use a room at the hospital or UCL. National event – somewhere like RCGP.** - Are there any other resources used that should be considered? **Travel, accommodation, advertising the course** |
| 1. If you were producing a series of 4 1-hour lectures on AMS for interns in a hospital,  - How many staff would you employ to produce the lectures? **Ideally 4 people but could be one person with appropriate skills** - What experience would you employ (e.g. AMS consultant, pharmacist, clinical lecturer, other)? **E.g. Consultant microbiologist/infectious diseases or registrar, AMS pharmacist.** - How many days would you plan for each person to spend developing it? **2-3 days** - Where would you hold such an event? **At the hospital in one of the training rooms** - Are there any other resources used that should be considered? |
| 1. If you were producing a 1-hour lecture for consultants and junior doctors on the updated AMS guidelines in a hospital,  - How many staff would you employ to produce the lectures? **1-3 Maybe one to develop content and others to review** - What experience would you employ (e.g. AMS consultant, pharmacist, clinical lecturer, other)? **Someone from the AMS Committee (Consultant or pharmacist)** - How many days would you plan for each person to spend developing it? **1 day for content developer, 1 hour per reviewer** |
| 1. If you were producing an e-learning course on AMS for consultants and junior doctors in a hospital,   **(I’m making the assumption that the course is e.g. 30-60 mins and for use locally not nationally)**   - How many staff would you employ to produce the e-learning course? – **1 to develop, 1-3 people to review** - What experience would you employ (e.g. AMS consultant, pharmacist, clinical lecturer, software programmer, IT expert, other)? **Consultant microbiologist/infectious diseases and/or pharmacist and then colleagues to review materials (registrar, Consultants) plus IT expert to format materials in line with hospital training** - How many days would you plan for each person to spend developing it? **1 day to develop, 1-2 hours per person to review** - Are there any other resources used that should be considered? **If this was to be used regionally / nationally would require additional input to develop high quality slides.** |
| 1. If you were updating an electronic prescribing system for antibiotics based on updated national guidelines in a hospital,   **Totally depends on the complexity of the e-prescribing system and the extent of IT support that is available locally. This could be a very significant task involving input from clinicians and software engineers. At UCLH I would anticipate you would need a meeting among the Consultant microbiologists to agree the change and then support from the software engineers to enact it. I can’t estimate the time involved for the latter.**   - How many staff and what staff would you employ to update it? - How many hours or days would you plan for it to take them to update it per person? - Are there any other resources used that should be considered? |
| 1. If you were planning an intervention where pharmacists or an AMS consultant did an audit and feedback on AMS prescribing behavior,  - Who would you organize to do the audit and feedback? **Depends on whether this is using electronic data or manual data collection. Electronic data capture requires an analyst, Consultant microbiologist/pharmacist to review the data and then provision of feedback so a series of team meetings. Manual data collection requires extraction of data usually by pharmacists and then presentation of that data back to clinicians.** - How many hours per week would you expect someone to spend on audit, evaluation and feedback? **If the system is set up to extract the data (very rare), someone could spend 4 hours doing this per week. If prospective data collection was required the time commitment would be much more extensive e.g. one month collecting data on the wards and them analyst and pharmacist time to convert this information into feedback.** - Are there any other resources used that should be considered? |
| 1. Imagine you were planning counselling interviews (CIs). This activity was carried out by a group of clinical experts who were selected by the Institutional Programme for the Optimization of Antimicrobial Treatment (PRIOAM) operations team, and included seven ID specialists, six critical-care specialists and four paediatricians. PRIOAM advisors were selected from local leaders in the management of patients with infectious diseases in each area. Each advisor conducted CIs in his/her area of responsibility, concerning a specific antimicrobial treatment randomly selected by the pharmacy service. A CI is conducted with the prescribing physician.  - How much time would you plan for in the development of a structured questionnaire for use in each interview (in hours or days)? **1 day plus time for the document to be peer reviewed by colleagues** - **How much time would you plan for in the preparation of each interview (in minutes or hours)? Person would need to familiarise themselves with the case e.g. 20 mins** - For how long would you plan each interview to last (in minutes or hours)? **15-30 minutes** - Are there any other resources used that should be considered? |
| 1. If you were planning for an AMS healthcare professional to attend ward rounds to provide advice on AMS,  - Which healthcare professional would you select for this task? (e.g. infectious disease consultant, AMS specialist nurse) **Consultant Microbiologist** - How many hours a week would you plan for the healthcare professional to spend each week on the ward rounds? **4 hours per week** - Are there any other resources used that should be considered? |
| 1. Across all the studies reported in Table 2, is there a difference in scope in the guideline development that may explain different levels of resource use required? Please could you try to describe different guideline development scopes and list these in Table 3? Possible examples have been listed below. Please could you categorise each study in Table 2 according to one of the scopes you have identified?   Possible examples written by us, which may be inappropriate (please use your own descriptions):  A: Development of AMS guidelines from scratch, conducting a systematic review of the literature, and a consultation process to decide on the content of the guidelines across different drugs and diseases/conditions.  B: Development of AMS guidelines from scratch, conducting a systematic review of the literature, and a consultation process to decide on the content of the guidelines for a specific drug in a specific condition.  C: Adaptation of local hospital guidelines to account for changes in national guidelines across different drugs and diseases/conditions.  D: Adaptation of local hospital guidelines to account for changes in national guidelines for a specific drug in a specific condition.  E: Editing/updating hospital guidance to account for a specific change in recommendation across the hospital guidelines.  **I don’t think any of these guidelines were developed from scratch. For me the key difference is whether they are developing hospital wide guidelines covering every possible condition / different types of antibiotic use e.g. treatment and prophylaxis or if they are focused on a specific infection or drug e.g. linezolid. The latter would be a lot less work.**  **Some of the studies are mainly desk-based where experts review and update guidelines, whereas others involve extensive consultation with a broader group of physicians and surgeons and I would anticipate this would require more intensive effort.** |

#### A2.2.1.1 Intervention component activities

Included studies evaluated multi-component interventions. The component activities were costed. The following activities were included in at least 5 studies: in-person education sessions, guideline development and publications, audit and feedback, educational interview, and ward round/ward presence. Other activities included in fewer studies were: e-learning, e-prescribing software, electronic administrative system order set, ongoing pharmacy support, monitoring, written bulletins and posters, phone calls, order sheet, carrying out of blood cultures, isolation, and incentives.

Only the studies included in the effectiveness analysis were included in the costing exercise. The activities costed for each study are presented in Table C2.

Key resource utilisation components of each activity were identified and costed. For instance, in-person education sessions included the number of man-hours required for producing on-site education sessions, the quantity of personnel involved in creating these sessions, the job role of individuals engaged in both production and delivery of on-site education sessions, the duration of each individual on-site education session, the total number of on-site education sessions, the cumulative man-hours dedicated to delivering education, and the total count of attendees (along with their specific areas of expertise) participating in the on-site education sessions.

*Table C2: List of intervention activities costed in each study*

| **No** | **Included studies** | **L** | **G** | **A&F** | **EI** | **WR** | **EL** | **I** | **EP** | **EA** | **PS** | **BP** | **OS** | **BC** | **I** |
| --- | --- | --- | --- | --- | --- | --- | --- | --- | --- | --- | --- | --- | --- | --- | --- |
| 1 | **Carrara 2022** | * | * | * |  |  |  | * |  |  |  |  |  |  |  |
| 2 | **Corcoine 2022** | * | * |  |  |  |  |  |  |  |  |  |  |  |  |
| 3 | **Smoke 2022** | * |  |  |  |  |  |  |  |  |  |  |  |  |  |
| 4 | **Liu 2021** | * |  | * |  |  |  | * |  |  |  |  |  |  |  |
| 5 | **Alvarez-Marin 2021** |  | * | * | * |  |  |  | * |  |  |  |  |  |  |
| 6 | **Seddik 2021** | * |  |  |  |  |  |  |  | * |  |  |  |  |  |
| 7 | **Du 2020** | * |  | * |  | * |  |  |  |  |  |  |  |  |  |
| 8 | **Gardiner_2020** | * | * |  |  |  |  |  |  |  | * |  |  |  |  |
| 9 | **Kjaersgaard_A 019** | * | * | * |  | * | * |  |  |  |  |  |  |  |  |
| 10 | **Garcia-Martinez 2016** |  | * |  | * |  |  |  |  |  |  |  |  |  |  |
| 11 | **Molina_2017** |  | * |  | * |  |  |  |  |  |  |  |  |  |  |
| 12 | **Molina_2019** |  | * |  | * |  |  |  |  |  |  |  |  |  |  |
| 13 | **Chang 2017** | * |  |  |  |  |  |  |  |  |  |  |  |  |  |
| 14 | **Tedeschi 2017** | * | * |  |  | * |  |  |  |  |  |  |  |  |  |
| 15 | **Gardiner_2018** | * | * |  |  |  |  |  |  |  |  | * |  |  |  |
| 16 | **Adachi 1997** |  | * | * |  |  |  |  |  |  |  |  | * |  |  |
| 17 | **Belliveau 1996** | * |  | * |  |  |  |  |  |  |  |  |  |  |  |
| 18 | **Hadi 2008** | * | * |  |  |  |  |  |  |  |  |  |  | * |  |
| 19 | **Knudsen 2014** | * | * |  |  | * |  |  |  |  |  |  |  |  | * |
| 20 | **Lee 2014** | * |  | * |  |  |  |  |  |  |  |  |  |  |  |
| 21 | **Liebowitz 2008** | * | * |  |  | * |  |  |  |  |  |  |  |  |  |
| 22 | **Willemsen 2010** | * | * | * |  |  |  |  |  |  |  |  |  |  |  |
| 23 | **Tangden 2011** | * | * |  |  |  |  |  |  |  |  |  |  |  |  |
| 24 | **Skaer 1993** |  |  | * |  | * |  |  |  |  |  |  |  |  |  |
| 25 | **Schwartz_2007** | * |  |  |  |  |  |  |  |  |  |  |  |  |  |
| 26 | **Popovski 2015** | * | * |  |  |  |  |  |  |  |  |  |  |  |  |

L (group events, lectures, seminar, presentations), G (guideline), A&F (Audit & Feedback), EI (Educational interviews), WR (Ward Round), EL (E-learning), I (Incentives), PS (E-prescribing software), EA (Electronic administrative system order set), PS (Ongoing pharmacy support), BP(Written bulletins and posters), OS(Order sheet), BC (Carrying out of blood cultures), I (Isolation)

#### A2.2.1.2 Imputation of education session items

There were missing data after data extraction and receipt of author responses. The number of education sessions delivered, and the number of consultants, junior doctors, nurses and pharmacists attending training sessions were imputed in some cases. Different methods were used. The assumptions made for each study are reported in Table C3. Often, the method started with number of hospital beds included in the hospital departments where the intervention was evaluated. Where the study did not report the number of beds or only gave information on the entire hospital, the hospital beds in a quoted hospital or hospital department was researched on the internet.

The ratio of junior doctors to hospital beds in England, the ratio of consultants to junior doctors in England, the ratio of hospital nurses to junior doctors and the and assumed number of 30 beds per ward were used to determine

- The number of education sessions if related to the number of wards
- The number of consultants, junior doctors, nurses and pharmacists who may be invited to attend an education session

*Table C3: Resource use assumptions made by included study (excluding type of healthcare professional involved in delivering education and training activities)*

| **Included studies** | **Assumptions** |
| --- | --- |
| **Carrara 2022** | **Education:**  None  **Guidelines:**  None  **Audit and feedback:**  17 audits (Author reported audit every 7-14 days in first 3 months, then once every month for 6 months in paper; every 7 days in first 3 months, then once every month for 6 months in AR*)  **Ward rounds/presence on wards:**  50%FTE for 3 months, then 20%FTE for 9 months for ID specialist  **Incentives:**  Only 2 consultant hours costed for incentives management |
| **Corcoine 2022** | **Education:**  Number of follow-up meetings assumed to be the same as initial meetings  Follow-up meetings assumed to be 30 mins  Ratio of general and acute beds to total beds assumed  Ratio of Consultants to Junior doctors assumed  Number of beds per ward assumed  (Author reported doctors per ward in AR*)  **Guidelines:**  None |
| **Smoke 2022** | **Education:**  Ratio of beds to Junior doctors assumed  (Author reported number of Junior doctors in AR*) |
| **Liu 2021** | **Education:**  1 lecture per ward  Number of beds per ward assumed  Length of education 45 mins in base case  Ratio of beds to Junior doctors assumed  Ratio of Consultants to Junior doctors assumed  60% of doctors attended a session in the base case (due to one-off nature of session)  Pharmacist spent 1 day preparing education session (other included studies and expert opinion)  **Audit and feedback:**  Duration of monthly audit the same as for Kaesrgaard, proportionally increased according to bed size and divided by 2 (double the beds may not mean double the cases audited)  **Incentives:**  Only 3 consultant hours costed for incentives management |
| **Alvarez-Marin 2021** | **Guidelines:**  3 staff for each of 7 hospitals, spending a day each. (Author refers to each local research team)  **Audit and feedback:**  8 hours per annual audit for each of the 7 hospitals  **Educational interview:**  None  **E-prescribing software:**  4 people spending a day each editing the software |
| **Seddik 2021** | **Education:**  Length of education 45 mins in base case  Pharmacist spent 1 day preparing education session (other included studies and expert opinion)  Ratio of beds to Junior doctors assumed  Ratio of Consultants to Junior doctors assumed  90% of doctors attended a session in the base case (authors reported that most doctors attended a session)  **Electronic system ordering set:**  4 people spending a day each editing the software |
| **Du 2020** | **Education:**  Doctors attended 4 sessions each  One session per month given at 15 mins each for 18 month duration of intervention  (Authors stated that frequent physician training given in paper)  Pharmacist spent 1 day preparing education session (other included studies and expert opinion)  Ratio of beds to Junior doctors assumed  Ratio of Consultants to Junior doctors assumed  **Audit and feedback:**  1 hour a week in the base case  **Ward rounds/presence on wards:**  Number of beds per ward assumed (4 hours per ward) |
| **Gardiner_2020** | **Education:**  1 education session per ward  Number of beds per ward assumed  Consultant spent 1 day preparing education session (other included studies and expert opinion)  Ratio of beds to Junior doctors assumed  Ratio of Consultants to Junior doctors assumed  60% of doctors attended a session in the base case (due to one-off nature of session)  836 beds identified from website (~800 reported in paper)  **Guidelines:**  Similar description to Popovski. Same estimate used.  **Ongoing pharmacy support:**  Number of beds per ward assumed (half an hour per ward per week)  **Written bulletins and posters:**  2 staff spending a day each |
| **Kjaersgaard_A 019** | **Education:**  1 session assumed for each of the 5 departments in each of the 2 hospitals reported in the paper. (6 smaller sessions for nurses reported)  Ratio of Consultants to Junior doctors assumed  (Author reported number of doctors who knew about guideline- assumed these all attended a session)  10 nurses attended each of the 6 smaller sessions reported to have taken place for nurses.  **Audit and feedback:**  8 hours by 2 people per monthly audit  400 hours spent developing the audit system (600-800 hours in total on audit and feedback reported by the author in AR*)  **Ward rounds/presence on wards:**  Number of beds per ward assumed (4 hours per ward)  **E-learning:**  None |
| **Garcia-Martinez 2016** | **Guidelines:**  20 days to conduct relatively short systematic review, and 4 committee members spending 1 day.  **Educational interview:**  10 minute interviews (same as Molina and Alvarez Marin)  Number of interviews assumed to be the average of the number of interviews per bed in the Alvarez-Marin and Molina 2017, and then adjusted for bed size |
| **Molina_2017** | **Guidelines:**  15 man-days to produce review using a multi-disciplinary team (Infectious Diseases physicians, Microbiologists, Pharmacists, Intensive Care physicians, Paediatricians and Preventivists). (development of consensual clinical guidelines for MDR bacteria and Candida spp.)  **Educational interview:**  None |
| **Molina_2019** | **Guidelines:**  15 man-days to produce review using a multi-disciplinary team (Infectious Diseases physicians, Microbiologists, Pharmacists, Intensive Care physicians, Paediatricians and Preventivists). (development of consensual clinical guidelines for common infectious syndromes in patients with cancer)  **Educational interview:**  None |
| **Chang 2017** | **Education:**  Length of education 45 mins in base case  Pharmacist and consultant spent 1 day each preparing education session (other included studies and expert opinion)  Ratio of beds to Junior doctors assumed  Ratio of Consultants to Junior doctors assumed |
| **Tedeschi 2017** | **Education:**  (Author reported that 1 topic was repeated twice a month each month for 3 months, and then changed to next topic. There were 5 topics)  Length of education 45 mins in base case  Consultant spent 1 day each preparing education session for each topic (other included studies and expert opinion)  Ratio of beds to Junior doctors assumed  Ratio of Consultants to Junior doctors assumed  60% of nurses attended a session in the base case (due to time constraints getting all nurses to attend)  Ratio of nurses to Junior doctors assumed  **Guidelines:**  Similar to Willemsen description. Same estimates of 4 staff spending 2 hours per guideline review. Guideline review occurred 5 times. |
| **Gardiner_2018** | **Education:**  1 education session per ward  Number of beds per ward assumed  10 minutes per education session as in Gardiner 2020  Consultant spent 1 day preparing education session (other included studies and expert opinion)  Ratio of beds to Junior doctors assumed  Ratio of Consultants to Junior doctors assumed  60% of doctors attended a session in the base case (due to one-off nature of session)  836 beds identified from website (~800 reported in Gardiner 2020)  **Guidelines:**  Similar description to Popovski. Same estimate used.  **Electronic system ordering set:**  4 people spending a day each editing the software  **Ongoing pharmacy support:**  Number of beds per ward assumed (half an hour per ward per week)  **Written bulletins and posters:**  2 staff spending a day each |
| **Adachi 1997** | **Guidelines:**  Similar description to Popovski. Same estimate used.    **Audit and feedback:**  8 hours per monthly audit  **Paper based order sheet update:**  4 people spending a day each |
| **Belliveau 1996** | **Education:**  5 education sessions required to cover 26 pharmacists, they can’t all attend at the same time  20 mins per education session  4 hours to produce education session for pharmacists  Assumption of pharmacist to bed ratio  **Audit and feedback:**  1 hour per audit proportionally adjusted by bed size relative to Du |
| **Hadi 2008** | **Education:**  Number of beds per ward assumed  (Authors reported 5 wards were involved)  **Guidelines:**  1 day per person. (author reported 7 departments involved- assumption 1 representative per department)  **Carrying out blood cultures:**  £50 a culture |
| **Knudsen 2014** | **Education:**  Ratio of beds to Junior doctors assumed  Ratio of Consultants to Junior doctors assumed  60% of doctors attended a session in the base case (due to one-off nature of session)  **Guidelines:**  None  **Ward rounds/presence on wards:**  Number of beds per ward assumed (4 hours per ward)  **Isolation:**  Additional cost of isolation over hospital stay: £500 |
| **Lee 2014** | **Education:**  None  **Audit and feedback:**  None |
| **Liebowitz 2008** | **Education:**  6 lectures (author reported different lectures at different forums and new staff groups)  Length of education 45 mins in base case  Consultant spent 1 day preparing education session (other included studies and expert opinion)  Ratio of beds to Junior doctors assumed  Ratio of Consultants to Junior doctors assumed  **Guidelines:**  Similar description to Popovski. Same estimate used.  **Ward rounds/presence on wards:**  Number of beds per ward assumed (4 hours per ward) |
| **Willemsen 2010** | **Education:**  1 lecture per ward  Number of beds per ward assumed  **Guidelines:**  None  **Audit and feedback:**  1 hour per audit proportionally adjusted by bed size relative to Du |
| **Tangden 2011** | **Education:**  1 lecture per ward  Number of beds per ward assumed  (Author reported the number of consultants and junior doctors per ward invited to attend education sessions)  60% of doctors attended a session in the base case (due to one-off nature of session)  **Guidelines:**  7 man-days involved. (author reported that 2 pharmacists worked with a local group network) |
| **Skaer 1993** | **Audit and feedback:**  1 hour per audit proportionally adjusted by bed size relative to Du |
| **Schwartz_2007** | **Education:**  (Author reported that junior doctors attend in groups of 5, and attended 4 sessions each. Plus 1 session.)  Ratio of nurses to doctors assumed |
| **Popovski 2015** | **Education:**  1 lecture per ward (author stated 1 each month)  Number of beds per ward assumed  Length of education 45 mins in base case  Consultant spent 1 day preparing education session (other included studies and expert opinion)  500 beds. Surgical department only, Charlton campus. Max 600 from website.  Ratio of beds to Junior doctors assumed  Ratio of Consultants to Junior doctors assumed  60% of doctors attended a session in the base case (due to one-off nature of session)  **Guidelines:**  None |

#### A2.2.1.3 Assumptions and scenarios

Assumptions were made for resource use for other component activities, such as the number of hours spent adapting a guideline for the local hospital or spent conducting an audit and providing feedback. The full list of assumptions excluding job title assumptions are listed by study in Table C3.

The significance of the uncertainty in assumptions made was evaluated by running analyses with alternative assumptions. The base case and scenario values are presented in Table C4. The ranks of each study by cost were obtained for each scenario (see Table C5).

*Table C4: Healthcare professional and hospital bed assumptions and sources*

| **Variable** | **Estimate** | **Source** |
| --- | --- | --- |
| Number of hospital doctors  Number of hospital consultants  Number of junior doctors in England  Consultants as a % of hospital doctors | 128,000  53,000  75,000  41.4% | Key facts and figures about the NHS. The King’s Fund. |
| Number of hospital nurses and midwives  Number of midwives  Number of hospital nurses      Ratio of hospital nurses to hospital doctors | 360,000    22,391  337,609      2.64 | The NHS workforce in numbers. The Nuffield Trust.  Midwife number slumps. Royal College of Midwives.  Imputed from above + ‘Health at a Glance: 2019 Indicators, OECD’ |
| Ratio of pharmacists to beds | 0.066 (0.043–0.088) wte | Borthwick M *et al*. |
| General and acute beds as a percentage of total beds in England | 71.5% | NHS hospital beds data analysis. BMA. |
| Number of beds per ward (base case)    Number of beds per ward (scenario analysis) | 30    20 | Assumption    Assumption |

*Table C5: List of scenarios defined.*

|  | **Scenario analyses** | **Base case** | **Scenario** |
| --- | --- | --- | --- |
| 1 | Duration of each session | 0.75 | 1 |
| 2 | Duration of each session | 0.75 | 0.5 |
| 3 | Number of beds per ward | 30 | 20 |
| 4 | Ratio of general and acute beds to total beds | 0.715 | 0.8 |
| 5 | Ratio of general and acute beds to total beds | 0.715 | 0.6 |
| 6 | Proportion of hospital doctors who are consultants | 0.414 | 0.3 |
| 7 | Junior doctors per bed | 0.47 | 0.35 |
| 8 | Size factor | 240 | 500 |
| 9 | Guideline hours per person | 7.5 | 15 |
| 10 | Audit & Feedback (hours per week) | 1 | 2 |
| 11 | Audit & Feedback (hours per annual audit) | 8 | 16 |
| 12 | Ongoing pharmacy support | 52 | 104 |
| 13 | % of doctors attending an education session | 0.6 | 0.7 |
| 14 | % of doctors attending an education session | 0.6 | 0.5 |
| 15 | % of nurses attending an education session | 0.6 | 0.7 |
| 16 | Excluding guideline intervention cost |  | 0 |

### 2 Unit costs

Almost all the cost of interventions was staff time. The cost of 1 hour of time of a healthcare professional in the NHS is reported in Table C6.

*Table C6: Unit cost values and sources*

| **Variable** | **Estimate (GBP)** |
| --- | --- |
| Consultant (1 hour) | 144 |
| Junior doctor (1 hour) | 56.3 |
| Nurse (1 hour) | 63 |
| Pharmacist (scientist) (1 hour) | 64 |
| Source: Jones *et al*. | |

### 3 Size factor adjustment

The effectiveness estimates for each study are standardised per patient or per number of patient-bed days. In contrast, the total cost of delivering the component activities of each intervention within each study depends on the number of wards, departments, hospitals across which the intervention was evaluated. The number of hospital beds included in the areas within which the intervention was evaluated was used as the measure of hospital size. In the base case, the study size factor was calculated as


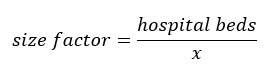


where is a standardised number of beds. In the base case the standardised number was set at 240. 240 represents a 240-bed hospital or intervention area. In scenario analysis this was increased to 500. The size factor can have a significant effect where there are fixed costs independent of size and where studies target different percentages of healthcare professionals within the hospital.

All the costs that may vary by size were standardised by dividing by the size factor.

### 4 GLM regression analysis

The incremental costs associated with the behaviour change characteristics were assessed using regression analysis. To ensure that cost estimates for all the explanatory variables included in the effectiveness analyses were produced, the full set of behaviour change characteristics in each category (BCT, BCW, mode of delivery, context) were included in the cost regressions where possible. Interaction terms were not considered due to the small data set.

The following generalised linear models were considered: a Gamma distribution with an inverse link function; a Gamma distribution with an identity link function. And a gaussian distribution with an identity link function. A Gamma distribution is often selected in the analysis of cost data due to the possibility of non-negative and skew mean linear predictor estimates. Of the models that could estimate a set of coefficients for the selected explanatory variables, the model with the lowest AIC was chosen.

A statistically significant result was one that was considered to have a p-value < 0.05.

### 5 Cost-effectiveness

The predicted cost and effect values from the regression and meta-regression analyses were obtained and plotted on scatterplots for each category of behaviour change characteristics (BCT, BCW, mode of delivery, context). A negative effect estimate represented an effective intervention. The effect estimates were therefore transformed to be positive so that a positive value represented an effective intervention.

Due to the fact that there are multiple behaviour change characteristics, negative cost and effect estimates for specific characteristics could be estimated by the statistical model due to association with other characteristics.

Each behaviour change characteristic was represented by a capital letter in the scatterplot, the cost-effectiveness plane. In the cost-effectiveness plane, the North-East quadrant is associated with greater effectiveness but also greater cost. The South-East quadrant is associated with greater effectiveness and lower cost. The strength of evidence effectiveness and cost difference association (positive or negative) were simply represented using differently coloured letters for different combinations of p-values for cost and effect coefficients.
