# Supplementary material for: A systematic review and meta-analysis to identify behavioural content and active ingredients of antimicrobial stewardship education and training interventions in hospital-based care settings
Source: Antimicrob Resist Infect Control. 2025 Dec 18;15:10. doi: 10.1186/s13756-025-01660-0 (PMC12829054; doi:10.1186/s13756-025-01660-0)
Supplement: Supplementary file 3 — Supplementary Material 3. [file 13756_2025_1660_MOESM3_ESM.docx]

# Supplementary file 3

# Health Economics Results

## 1 Study costs

The cost estimates for the intervention in each study are presented in Table C7. There is considerable variation in cost estimates with a few interventions associated with very high cost values. The study by Kjaersgaard et al, in 2019 has the highest cost, with the cost of £1,046,316 and the study by Belliveau in 1996 had the lowest cost of £8,323. The distribution of intervention costs is presented in Figure C1A. The most common activity costed was Education sessions, and its distribution is also presented in Figure C1B.

Figure C1: Histograms (A) Total cost of interventions, (B) Education session training costs


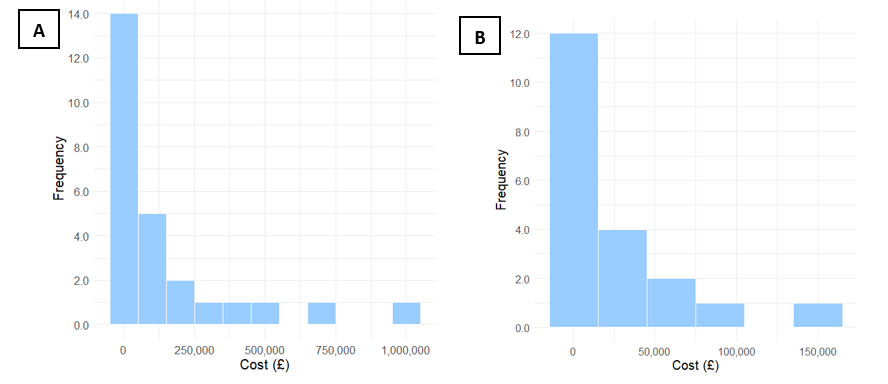


*Table C7: cost of intervention for studies and ranked for scenarios*

| **Study ID** | **Cost (£) (ranked)** | **Base-case**  **rank** | **Ranking scenarios** | | | | | | | | | | | | | | | |
| --- | --- | --- | --- | --- | --- | --- | --- | --- | --- | --- | --- | --- | --- | --- | --- | --- | --- | --- |
| **Sort by base-case rank** |  |  | **1** | **2** | **3** | **4** | **5** | **6** | **7** | **8** | **9** | **10** | **11** | **12** | **13** | **14** | **15** | **16** |
| Kjaersgaard_A 019 | 1,046,316 | 1 | 1 | 1 | 1 | 1 | 1 | 1 | 1 | 1 | 1 | 1 | 1 | 1 | 1 | 1 | 1 | 1 |
| Tedeschi 2017 | 692,845 | 2 | 2 | 2 | 2 | 2 | 2 | 2 | 2 | 2 | 2 | 2 | 2 | 2 | 2 | 2 | 2 | 2 |
| Knudsen 2014 | 456,36 | 3 | 3 | 3 | 3 | 3 | 3 | 3 | 3 | 3 | 3 | 3 | 3 | 3 | 3 | 3 | 3 | 3 |
| Liebowitz 2008 | 352,452 | 4 | 4 | 4 | 4 | 4 | 4 | 4 | 4 | 4 | 4 | 4 | 4 | 4 | 4 | 4 | 4 | 4 |
| Du 2020 | 346,177 | 5 | 5 | 5 | 5 | 5 | 5 | 5 | 5 | 5 | 5 | 5 | 5 | 5 | 5 | 5 | 5 | 5 |
| Skaer 1993 | 168,990 | 6 | 6 | 6 | 6 | 6 | 6 | 6 | 6 | 6 | 6 | 6 | 6 | 6 | 6 | 6 | 6 | 6 |
| Carrara 2022 | 166,231 | 7 | 7 | 7 | 7 | 7 | 7 | 7 | 7 | 7 | 7 | 7 | 7 | 7 | 7 | 7 | 7 | 7 |
| Hadi 2008 | 113,593 | 8 | 8 | 8 | 8 | 8 | 8 | 8 | 8 | 8 | 8 | 9 | 8 | 8 | 8 | 8 | 8 | 8 |
| Liu 2021 | 66,411 | 9 | 9 | 9 | 9 | 10 | 9 | 9 | 9 | 11 | 9 | 10 | 9 | 9 | 9 | 9 | 9 | 9 |
| Willemsen 2010 | 63,340 | 10 | 10 | 10 | 10 | 9 | 10 | 10 | 10 | 12 | 12 | 8 | 10 | 10 | 10 | 10 | 10 | 10 |
| Molina_2019 | 54,616 | 11 | 11 | 11 | 11 | 11 | 11 | 11 | 11 | 10 | 13 | 11 | 11 | 11 | 11 | 11 | 11 | 12 |
| Lee 2014 | 53,567 | 12 | 12 | 12 | 12 | 12 | 12 | 12 | 12 | 9 | 14 | 12 | 12 | 12 | 12 | 12 | 12 | 11 |
| Alvarez-Marin 2021 | 41,583 | 13 | 13 | 13 | 13 | 13 | 13 | 13 | 13 | 14 | 11 | 13 | 13 | 13 | 13 | 13 | 13 | 15 |
| Garcia-Martinez 2016 | 39,418 | 14 | 14 | 14 | 14 | 14 | 14 | 14 | 14 | 15 | 10 | 14 | 14 | 14 | 14 | 14 | 14 | 21 |
| Molina_2017 | 35,292 | 15 | 15 | 15 | 15 | 15 | 15 | 15 | 15 | 13 | 15 | 15 | 15 | 16 | 15 | 15 | 15 | 14 |
| Schwartz_2007 | 27,462 | 16 | 17 | 16 | 17 | 16 | 16 | 16 | 16 | 18 | 19 | 16 | 16 | 17 | 16 | 16 | 16 | 13 |
| Gardiner_2020 | 24,252 | 17 | 18 | 17 | 16 | 17 | 17 | 17 | 17 | 16 | 17 | 17 | 17 | 15 | 17 | 17 | 17 | 16 |
| Popovski 2015 | 23,864 | 18 | 16 | 20 | 18 | 18 | 18 | 20 | 20 | 17 | 18 | 18 | 18 | 18 | 18 | 19 | 18 | 17 |
| Tangden 2011 | 23,280 | 19 | 19 | 18 | 20 | 19 | 19 | 18 | 18 | 22 | 16 | 19 | 19 | 19 | 19 | 18 | 19 | 19 |
| Corcoine 2022 | 22,513 | 20 | 20 | 19 | 19 | 20 | 20 | 19 | 19 | 19 | 20 | 20 | 20 | 20 | 20 | 20 | 20 | 22 |
| Seddik 2021 | 15,747 | 21 | 21 | 22 | 21 | 21 | 21 | 21 | 21 | 21 | 22 | 21 | 21 | 21 | 21 | 21 | 21 | 18 |
| Chang 2017 | 14,895 | 22 | 22 | 24 | 22 | 22 | 22 | 22 | 22 | 20 | 24 | 23 | 22 | 22 | 22 | 22 | 22 | 20 |
| Gardiner_2018 | 11,911 | 23 | 23 | 21 | 23 | 23 | 23 | 23 | 23 | 24 | 21 | 24 | 23 | 23 | 23 | 24 | 23 | 25 |
| Smoke 2022 | 11,083 | 24 | 24 | 23 | 24 | 24 | 24 | 24 | 25 | 23 | 25 | 25 | 24 | 24 | 24 | 23 | 24 | 23 |
| Adachi 1997 | 9,944 | 25 | 25 | 25 | 25 | 25 | 25 | 25 | 24 | 25 | 23 | 26 | 25 | 25 | 25 | 25 | 25 | 26 |
| Belliveau 1996 | 8,323 | 26 | 26 | 26 | 26 | 26 | 26 | 26 | 26 | 26 | 26 | 22 | 26 | 26 | 26 | 26 | 26 | 24 |

## 2 Scenario analysis

The results of the scenario analyses are presented in Table C8. The rank of studies according to cost in the base case is presented, alongside the cost rank for each study for each scenario. Shaded cells indicate that the rank of the study has changed from the base case.

A few scenario analyses had no effect on study ranks. Scenario 8 (size factor), scenario 9 (guideline hours per person), and scenario 16 (exclusion of guideline intervention costs) had the greatest impact on study ranks.

## 3 Cost regression results

A Gaussian glm with identity link was selected for the BCT and BCW analyses. A Gamma glm with an inverse link was selected for the mode of delivery analysis. A Gamma glm with an identity link was selected for the context analysis.

Table C8 displays the predictive values of regression models applied to behavioural change techniques (BCTs), modes of delivery, behavioural change wheel factors, and context explanatory variables in the base case analysis. Discrepancy between current behaviour and goal, information about health consequences, credible source and restructuring the physical environment were the BCTs that had a statistically significant association with cost. Reducing bad practice was the only context variable to have a statistically significant association with cost. No mode of delivery or BCW factor had a statistically significant association with cost. This is likely due to the correspondence with component activities.

Regression results for scenarios 8 and 9 from Table C7 are reported in Tables C9 and C10.

*Table C8: Base case GLM regression results for BCT, Mode of delivery, BCW, and Context analyses*

| **Base-case** | | | | | |
| --- | --- | --- | --- | --- | --- |
| **BCT** | **Predicted (£)** | **Sig** | **Mode** | **Predicted (£)** | **Sig** |
| (Intercept) | 278,622 |  | (Intercept) | 52,654 | * |
| Goal | 504,520 |  | Print | 75,392 |  |
| Action | 339,972 |  | Telephone | 70,427 |  |
| Review | 217,682 |  | Ftf | 123,032 |  |
| Discrep | 32,990 | * |  |  |  |
| beh_cont | 197,474 |  | **BCW** |  |  |
| feed_beh | 168,451 |  | (Intercept) | 134665.6 |  |
| feed_out | 531,956 |  | Training | 145367.9 |  |
| supp_pract | -99,649 |  | Modelling | 49219.38 |  |
| Instruct | 275,571 |  | ResEnvironment | 173193.2 |  |
| info_health | 310,807 | ** | Incentivisation | 45176.5 |  |
| info_ante | 401,955 |  | Restriction | 34419.73 |  |
| Demo | -2,281 |  |  |  |  |
| Prompt | 606,488 |  | **Context** |  |  |
| beh_sub | 420,376 |  | (Intercept) | 381344.6 | ** |
| Cred | -9,744 | * | m_ward | 248948.2 |  |
| res_soc | 269,820 |  | doc_pre | 329435 |  |
| res_phys | 504,520 | * | act_reduc | 45392.03 | * |
| add_env | 339,972 |  |  |  |  |
| Sig: statistical significance codes: ** p-value < 0.01, * p-value < 0.05 | | | | | |

*Table C9: Scenario 8 GLM regression results for BCT, Mode of delivery, BCW, and Context analyses*

| **Scenario 8** | | | | | |
| --- | --- | --- | --- | --- | --- |
| **BCT** | **predicted** | **Sig** | **Mode** | **predicted** | **Sig** |
| (Intercept) | 48069.53 |  | (Intercept) | 97115.66 | * |
| goal | -47737.9 | * | Print | 139997.2 |  |
| action | -382351 |  | Telephone | 133351.1 |  |
| review | 18135.56 | . | Ftf | 226757.4 |  |
| discrep | 31333.13 |  |  |  |  |
| beh_cont | 15732.57 | . | **BCW** |  |  |
| feed_beh | 17376.41 | * | (Intercept) | 294898.3 |  |
| feed_out | -46700.6 | * | Training | 327086 |  |
| supp_pract | -242184 |  | Modelling | 85801.56 |  |
| instruct | 23089.68 |  | ResEnvironment | 319264.4 |  |
| info_health | 18939.82 | * | Incentivisation | 67055.14 |  |
| info_ante | 62482.43 |  | Restriction | 45987.58 |  |
| demo | -49464.8 | . |  |  |  |
| prompt | 46430 |  | **Context** |  |  |
| beh_sub | 30980.37 |  | (Intercept) | 795608.2 |  |
| cred | -66942.5 | * | m_ward | 465332.7 |  |
| res_soc | 514535.6 | . | doc_pre | 745378.7 |  |
| res_phys | 25723.21 |  | act_reduc | 79349.97 | . |
| add_env | 119743.3 |  |  |  |  |
| Sig: statistical significance codes: ** p-value < 0.01, * p-value < 0.05 | | | | | |

*Table C10: Scenario 9 GLM regression results for BCT, Mode of delivery, BCW, and Context analyses*

| **Scenario 9** | | | | | |
| --- | --- | --- | --- | --- | --- |
| **BCT** | **predicted** | **Sig** | **Mode** | **predicted** | **Sig** |
| (Intercept) | 45047.07 |  | (Intercept) | 55300.56 | * |
| goal | -45863.1 | * | Print | 79535.51 |  |
| action | -129082 | . | Telephone | 73887.99 |  |
| review | 16641.7 | . | Ftf | 126742.7 | . |
| discrep | 32935.91 |  |  |  |  |
| beh_cont | 15336.01 | . | **BCW** |  |  |
| feed_beh | 14694.65 | ** | (Intercept) | 132770.4 |  |
| feed_out | -31982.6 | * | Training | 134750.9 |  |
| supp_pract | -530786 | . | Modelling | 51114.55 |  |
| instruct | 21228.72 |  | ResEnvironment | 179940.3 |  |
| info_health | 14395.53 | ** | Incentivisation | 46563.39 |  |
| info_ante | 49741.35 |  | Restriction | 36070.35 |  |
| Demo | -32127.5 | * |  |  |  |
| Prompt | 40386.09 |  | **Context** |  |  |
| beh_sub | 21756.63 | . | (Intercept) | 378329.3 |  |
| Cred | -41814.8 | * | m_ward | 250300.4 |  |
| res_soc | 2624672 | * | doc_pre | 342724 |  |
| res_phys | 21388.09 | * | act_reduc | 51493.84 | . |
| add_env | -548546 | . |  |  |  |
| Sig: statistical significance codes: ** p-value < 0.01, * p-value < 0.05 | | | | | |

## 4 Cost-effectiveness results

The cost-effectiveness results are presented on cost-effectiveness planes. These are presented in Figure C2. Each behaviour change characteristic is labelled with a letter. The labelling by characteristic is presented in Table C11. Characteristics that associated with greater effectiveness and lower cost (those furthest South-East on the plane) may be more likely to be associated with cost-effective interventions. The strength of evidence is depicted by the colour of the letter label. ‘<0.2, <0.1’ indicates that the p-value for the effect results was <0.2 and the p-value for the cost results was <0.1.

The only factor that had a p-value for effect and cost <0.1 was face-to-face mode of delivery.

**Figure C2.** Cost-effectiveness panes planes for (A) BCT, (B) BCW, (C) Mode of delivery, and (D) Context variables


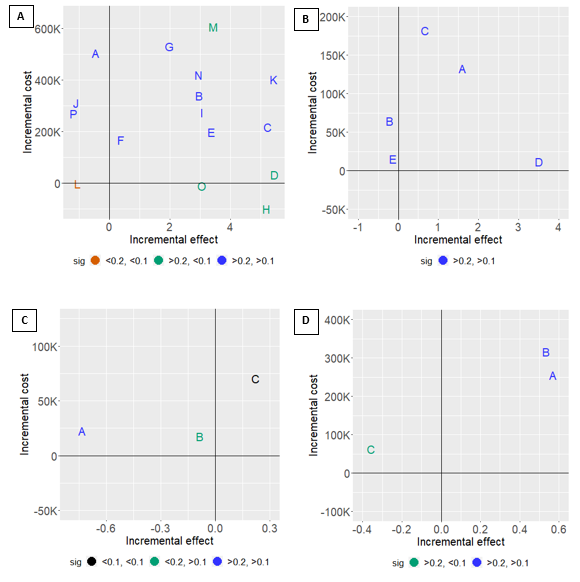


**Table C11.** Legend of letter labels in Figure C2 scatterplots

| **Plot A** | | | | **Plot B** | | **Plot C** | |
| --- | --- | --- | --- | --- | --- | --- | --- |
| A | Goal | I | info_ante | A | Training | A | print |
| B | Action | J | demo | B | Modelling | B | telephone |
| C | Review | L | prompt | C | ResEnvironment | C | ftf |
| D | Discrep | M | beh_sub | D | Incentivisation | **Plot D** | |
| E | beh_cont | N | cred | E | Restriction | A | m_ward |
| F | feed_beh | O | res_soc |  |  | B | doc_pre |
| G | supp_pract | P | res_phys |  |  | C | act_reduc |
| H | info_health | Q | add_env |  |  |  |  |
